# Untargeted metabolomics of human keratinocytes reveals the impact of exposure to 2,6-dichloro-1,4-benzoquinone and 2,6-dichloro-3-hydroxy-1,4-benzoquinone as emerging disinfection by-products

**DOI:** 10.1007/s11306-022-01935-2

**Published:** 2022-11-07

**Authors:** Dimitra G. Meintani, Theodoros G. Chatzimitakos, Athanasia I. Kasouni, Constantine D. Stalikas

**Affiliations:** 1 Laboratory of Analytical Chemistry, Department of Chemistry, University of Ioannina, 45110 Ioannina, Greece; 2 Laboratory of Biophysical Chemistry, Department of Biological Applications and Technologies, University of Ioannina, 45110 Ioannina, Greece

**Keywords:** 2,6-dichloro-1,4-benzoquinone, 2,6-dichloro-3-hydroxy-1,4-benzoquinone, Metabolomics, Disinfection byproducts, NMR, HaCaT

## Abstract

**Introduction:**

The 2,6-dichloro-1,4-benzoquinone (DCBQ) and its derivative 2,6-dichloro-3-hydroxy-1,4-benzoquinone (DCBQ-OH) are disinfection by-products (DBPs) and emerging pollutants in the environment. They are considered to be of particular importance as they have a high potential of toxicity and they are likely to be carcinogenic.

**Objectives:**

In this study, human epidermal keratinocyte cells (HaCaT) were exposed to the DCBQ and its derivative DCBQ-OH, at concentrations equivalent to their IC_20_ and IC_50_, and a study of the metabolic phenotype of cells was performed.

**Methods:**

The perturbations induced in cellular metabolites and their relative content were screened and evaluated through a metabolomic study, using 1H-NMR and MS spectroscopy.

**Results:**

Changes in the metabolic pathways of HaCaT at concentrations corresponding to IC_20_ and IC_50_ of DCBQ-OH involved the activation of cell membrane α-linolenic acid, biotin, and glutathione and deactivation of glycolysis/gluconeogenesis at IC_50_. The changes in metabolic pathways at IC_20_ and IC_50_ of DCBQ were associated with the activation of inositol phosphate, pertaining to the transfer of messages from the receptors of the membrane to the interior as well as with riboflavin. Deactivation of biotin metabolism was recorded, among others. The cells exposed to DCBQ exhibited a concentration-dependent decrease in saccharide concentrations. The concentration of steroids increased when cells were exposed to IC_20_ and decreased at IC_50_. Although both chemical factors stressed the cells, DCBQ led to the activation of transporting messages through phosphorylated derivatives of inositol.

**Conclusion:**

Our findings provided insights into the impact of the two DBPs on human keratinocytes. Both chemical factors induced energy production perturbations, oxidative stress, and membrane damage.

**Supplementary Information:**

The online version contains supplementary material available at 10.1007/s11306-022-01935-2.

## Introduction

Chlorination is the most frequent method for disinfection in both drinking water and swimming pools (Abbasnia et al., 2019) and it is performed by applying calcium hypochlorite, liquefied chlorine gas, sodium hypochlorite, or on-site chlorine generators (Hariganesh et al., 2020). When reacting with the natural organic and inorganic matter of water, disinfectants can form disinfection by-products (DBPs), with the aliphatic substances to be less toxic than aromatic ones (Tang et al., 2020). Halobenzoquinones (HBQs) are considered DBPs of particular importance as they have a high potential for toxicity and they are likely to be carcinogenic (Fu et al., 2017). They are formed after chlorination or chloramination of water (Kosaka et al., 2017). Hydroxylated HBQs are, substantially, less toxic than their parent compounds (Wang et al., 2014) and they are formed via hydrolysis (Hung et al., 2019) and ultra-violet irradiation (Görner & Von Sonntag, 2008). The 2,6-dichloro-1,4-benzoquinone (DCBQ) is the most commonly detected HBQ (Zhao et al., 2012), occurring at concentrations of 14.0–55.0 ng L^− 1^ (0.079–0.311 nM) (Qin et al., 2010) in drinking water and 27.0-300 ng L^− 1^ (0.15–1.69 nM) in pool waters (Wang et al., 2013a, b). The 2,6-dichloro-3-hydroxy-1,4-benzoquinone (DCBQ-OH), which is the main hydrolysis product of DCBQ, has been detected in drinking water distribution systems, at concentrations of up to 20.0 ng L^− 1^ (0.103 nM) (Hung et al., 2019).

In vitro studies are an invaluable source of information on human health, as the chemical damage and cytotoxicity can be evaluated with the use of cell cultures (Aragonès et al., 2017). Cells are handled easily, the testing of different compounds can be performed simultaneously and the chemical toxicity can be evaluated in a cost-effective way (Muñoz & Albores, 2010). DBPs produce adverse health effects, as it has already been demonstrated by many toxicological studies. Thus, it is necessary to thoroughly evaluate their effects after human exposure. Cultured human keratinocytes are employed to study the exposure of human skin to chemical factors (Li et al., 2021). The HaCaT is a monoclonal cell line that does not produce tumors. It is long-lived, thus allowing the continuous and uninterrupted study of cells, it does not require costly growth factors for its survival, making it ideal for the study of keratinocyte functions (Colombo et al., 2017).

Metabolomic screening is a promising tool to carry out the study of cell functions, allowing the identification and quantification of varying metabolites in biological samples. The identification of metabolites and the assessment of metabolic pathways provide valuable information about the toxicological effects and biological mechanisms of cells exposed to chemical factors (Oliveira et al., 2016). Nuclear Magnetic Resonance (NMR) spectroscopy has been playing a key role in decoding and understanding the metabolism and metabolic processes of exposed organisms and cells, for more than fifty years now (Williams et al., 1979; Winkel & Jans, 1990). It offers a variety of information, has the advantage of identifying multiple metabolites simultaneously and provides scientists with the possibility of quantifying them, through predictable and reproducible spectra (Wishart, 2019). Mass spectrometry (MS) has, also, been employed to identify unknown compounds and quantify known materials. Metabolomics based on MS has increased rapidly in the last decades due to advantages, such as high sensitivity and mass accuracy and reliable characterization of various biomolecules (Nagana Gowda & Djukovic, 2014). Taking into consideration that DCBQ and DCBQ-OH are predominant DBPs in the sanitization of swimming pool water (and not only), human epidermal keratinocytes were chosen in this study as the ideal in vitro model to examine the effect of the two chemical factors on the cellular viability and the metabolic phenotype of the exposed cells. The exposure concentrations for the metabolomic study were chosen based on the outcome of a cell viability study (Chatzimitakos et al., 2018). Then, the perturbations induced in cellular metabolites and their relative content were screened and evaluated through a metabolomic study using ^1^H-NMR and MS spectroscopy. The metabolic pathways were annotated and attributed to certain alterations of cells.

## Materials and methods

### Reagents

All chemicals used were of analytical grade. 2,2-Diphenyl-1-picrylhydrazyl (DPPH) was purchased from Aldrich (Steinheim, Germany). 2,6-dichloro-1,4-benzoquinone was purchased from Sigma Aldrich. HaCaT cells were used for the experiments. Dulbecco′s Modified Eagle Medium (DMEM), Fetal Bovine Serum (FBS), L-Glutamine, penicillin, and streptomycin were purchased from Gibco, Thermo Fischer Scientific (Australia). Trypsin-EDTA 0.5% was purchased from Gibco, Thermo Fischer Scientific (America), and crystal violet was purchased from Merck (Darmstadt, Germany). Cell culture plates were purchased from Greiner Bio-One GmbH (Frickenhausen, Germany). Cell culture multiwell plates were purchased from Thermo Scientific (Denmark). Deuterated water (D_2_O) and 3-(trimethylsilyl)-1-propionic-2, 2, 3, 3-d_4_ acid sodium salt (TSP) were obtained from Deutero GmbH (Kastellaun, Germany). Double distilled water (DDW) was used to prepare all solutions. Phosphate Buffer Saline (PBS) was prepared with NaCl: 137.0 mM, KCl: 2.7 mM, Na_2_HPO_4_: 10.0 mM, KH_2_PO_4_: 1.8 mM. All solutions for the cell cultures were sterilized by autoclaving at 121^o^C for 20 min, at 21 psi.

### Instrumentation

Instrumentation is described in detail in the Supplementary Information.

### Stability study of DCBQ

The stability of DCBQ in DMEM was performed by monitoring the transformation of DCBQ to DCBQ-OH, at room temperature and darkness. The DCBQ solutions at concentrations of 0.05 and 0.075 mM were prepared in DMEM and adjusted to pH = 5.0. The transformation into DCBQ-OH (%) was estimated after a 30 min period of exposure to DCBQ. The absorbance was measured at 530 nm (Görner & Von Sonntag, 2008) every 10 min, up until its value had reached a plateau, indicating that DCBQ was transformed to DCBQ-OH.

### Synthesis and stability study of DCBQ-OH

A quantity of 15.0 mg of DCBQ was dissolved in 1.0 mL of methanol and then DDW was added up to a final concentration of 3.0 mM. The solution was left to stand in the daylight for a period of 24 h. A DCBQ-OH solution of 1.0 mM was prepared, and the molecular absorbance (in the range of 400–600 nm) and NMR spectra were obtained.

### 2,2-Diphenyl-1-picryl-hydrazyl (DPPH) assay

A stock solution of DPPH at 6 × 10^− 5^ M was prepared in methanol. Solutions of DCBQ-OH in DDW and DCBQ in methanol were prepared at various concentrations (0.005–0.75 mM). An aliquot of 2.34 mL of DPPH and 0.66 mL of the solution of the compound tested were mixed and after stirring for 30 min the absorbance was measured at 517 nm. Control samples of DPPH were prepared with 2.34 mL of DPPH in DDW and methanol for DCBQ-OH and DCBQ, respectively. Blank samples containing 2.34 mL of DDW or methanol were prepared for DCBQ-OH or DCBQ, respectively. Spectrophotometric measurements were done and the % free radical scavenging activity was calculated (Chatzimitakos et al., 2016).

### Cultivation of HaCaT cells

HaCaT cells were stored in liquid nitrogen. The cell pellet was resuspended in a small volume of the cell culture medium DMEM. Cells were cultivated in cell culture dishes containing high-glucose DMEM, 1% penicillin, 1% streptomycin, 1% L-glutamine, and 10% FBS. Cultures were maintained in an incubator in a 5% CO_2_ atmosphere, at 37^o^C. Next, DMEM was discarded and cells were washed with PBS to remove dead cells. Cells were harvested by trypsinization and were put in cell culture multiwell plates for cell viability assay and metabolomic study. All experiments were done in a sterilized environment with laminar flow in α Euroclone Fume Hood.

### Cell viability assay

For the cell viability assay of DCBQ-OH, HaCaT cells were exposed to concentrations of 0.01–1.25 mM. The incubation was performed at 37^o^C in a humidified environment with 5% CO_2_, for 2, 6, 8, 12, and 24 h. The viability of cells at concentrations of 0.10 mM and 0.30 mM of DCBQ-OH was also evaluated for a 30-min exposure time. For the cell viability assay of DCBQ, HaCaT cells were exposed to concentrations of 0.01–0.30 mM for 30 min, in DMEM, at pH = 5.0. The cell viability assay is detailed in the Supplementary Information.

### Metabolomic assay

HaCaT cells were cultured and exposed to HBQs for the metabolomic assay. Cells were exposed to DCBQ-OH, at concentrations of 0.10 and 0.30 mM, for 24 h and to DCBQ, at concentrations of 0.05 and 0.075 mM, for 30 min. In both cases, the concentrations corresponded to the IC_20_ and IC_50_ of cells, respectively. Non-exposed samples were used as controls. Cells were harvested and metabolites were extracted. The extraction of metabolites was based on the Bligh-Dyer method (Ramiz & Soumen, 2019). The cell pellet was resuspended in 0.66 mL of DDW and 0.80 mL of methanol sequentially, at 4^o^C. Then, the pellet was subjected to three freeze-thaw cycles using liquid nitrogen and 1.6 mL of chloroform was added. The solution with the pellet was vortexed for 30 s and centrifuged at 5000 rpm, for 5 min. The supernatant was retracted, divided into two equal parts, transferred to Eppendorf vials, and was evaporated under a gentle stream of nitrogen. This procedure was repeated twice more, without the step of the freeze-thaw cycle and all the metabolites were collected in the Eppendorf vials. For ^1^H-NMR measurements, a portion of the residue was resuspended in 0.60 mL of deuterium oxide containing TSP (1 mM) as the internal standard. For the LC-HRMS study, the remaining portion of the residue was resuspended in 0.10 mL of acetonitrile.

### ^1^H-NMR spectra processing and relative quantification of metabolites

After obtaining the ^1^H-NMR spectra, the spectra were aligned using TSP (0.0 ppm), and the signal positions, in ppm, were input to the 1D NMR search engine of HMDB (Wishart et al., 2007, 2009, 2013, 2018) to realize identification of the metabolites. A list of metabolites for each sample was provided by the search engine. Verification of the metabolites was performed based on the obtained MS spectra. The exposure of HaCaT cells to DCBQ-OH and DCBQ led to alterations, which were evaluated by analyzing the metabolic pathways. MetaboAnalyst 5.0 was used for the pathway analysis employing the library of Homo Sapiens for HaCaT cells (Pang et al., 2021).

The variation in the metabolite relative contents was calculated by manually integrating the selected NMR signals using the TSP signal (δ = 0.00 ppm) as an internal standard. Every metabolite present in samples was assigned to ^1^H-NMR peaks, which were unique for the specific metabolite. The ratios of the integrals of metabolite NMR peaks to that of the TSP in the control sample were compared with the ratios of the same metabolites in the samples exposed to the chemical factors. The percentages of variations of the relative contents of metabolites were calculated. The reproducibility of the whole process for the metabolite identification and their relative quantification was ensured by assessing the ^1^H-NMR spectra, after performing the exposure experiments in cells, in triplicate. The relative standard deviation of the relative content did not exceed 7.5% for the metabolites studied.

### Statistical analysis

At first, data obtained from the study were examined, whether they are normally distributed, using Shapiro-Wilk test. Equality of variance was examined with the *F*-test. Where necessary, Mann-Whitney *U* test was applied to evaluate the statistical significance of the examined parameters, and differences were considered significant at p < 0.05 (n = 3).

## Results

### Synthesis and stability of DCBQ-OH and DCBQ

The DCBQ, at a concentration of 1.0 mM was transformed into the hydroxylated product, within 24 h, with the values of absorbance leveling off, at 530 nm (Fig. S1). At the same time, the pH of the solution of DCBQ-OH formed was 5.0.

The NMR spectrum of DCBQ-OH 1.0 mM showed two distinct peaks at 6.81 and 6.92 ppm, which correspond to the hydrogen on the benzoquinone ring and hydroxyl group, respectively (Fig. S2). The peak at 7.04 ppm in the DCBQ spectrum corresponds to the hydrogens of the benzoquinone ring (Fig. S3) (Wiley & Sons, 2021). The differences in the NMR spectra are attributed to the newly formed hydroxyl group of DCBQ-OH.

A stability study of DCBQ at concentrations of 0.05 and 0.075 mM, in DMEM of pH = 5.0, was realized by monitoring the transformation of DCBQ to DCBQ-OH. Taking into account that the pH of the solution is critical for cell viability and metabolomic study, the pH of DMEM varied to determine the pH value where the kinetic of transformation of DCBQ into its hydroxylated product is slow and cells remained viable. The conversion of DCBQ into DCBQ-OH was promoted at pH = 5.5 and 6.5 and kinetically favored over lower pH values. Even though at pH = 4.5 the DCBQ was slowly converted into DCBQ-OH, a 30-min exposure of cells to DMEM at this pH induced 20% death, (Fig. S4). At pH = 5.0, 54% and 40% of DCBQ at the low and high concentration was transformed into DCBQ-OH, in 30 min (Fig. S5).

### DPPH assay

The DCBQ-OH and DCBQ were evaluated for their potential to behave as free-radical scavengers, at concentrations of 0.005–0.75 mM. The experimental results showed that both chemical factors exhibited DPPH scavenging activity, which increased in a concentration-dependent manner. For both, DCBQ-OH and DCBQ at 0.20 mM, the % scavenging activity towards the DPPH radical reached a plateau, signifying that they both had reached their full potential as scavengers (Fig. S6).

### Cell viability assay

After 2, 6, 8, and 12 h of incubation at the tested concentrations of DCBQ-OH (0.01-1.0 mM), the HaCaT cells manifested viabilities greater than 65% (Fig. S7). The 24 h was selected as the appropriate time of exposure, as HaCaT cells showed a concentration-dependent decrease in cell viability in the range of 0.10–1.25 mM of DCBQ-OH, where significant metabolic perturbations occurred. The IC_50_ and IC_20_ values of DCBQ-OH were found to be 0.30 mM and 0.10 mM, respectively (Fig. S8). The control cells retained their viability throughout the 24 h.

On the other hand, the exposure of cells to DCBQ was performed in DMEM at pH = 5.0, for an exposure period of 30 min. These conditions were selected based on the stability study of DCBQ (Fig. S5). Under these conditions, only a limited transformation of DCBQ to DCBQ-OH took place. After a 30-min exposure of HaCaT cells to DCBQ, an abrupt decrease in their viability as a function of the concentration of DCBQ occurred. The IC_50_ and IC_20_ values of DCBQ were found to be 0.075 and 0.05 mM, respectively (Fig. S9). The viability of cells incubated in DMEM at pH = 5.0 and pH = 7.6, for 30 min were compared and the cells proved to retain their viability. Therefore, exposure to more acidic DMEM did not affect cell viability.

To determine whether the DCBQ-OH in samples affected the viability of cells, additional experiments with DCBQ-OH at 0.10 and 0.30 mM for 30-min exposure of cells, were performed. Cells exhibited viabilities greater than 95%, which suggests that DCBQ-OH did not contribute to death when cells were exposed to DCBQ. Therefore, it is reasonable to speculate that alterations in the viability of cells were attributed only to the presence of DCBQ.

As metabolic processes are closely related to the survivability of cells it is prudent to study the metabolome of cells exposed to DCBQ-OH and DCBQ and compare it with the metabolome of the non-exposed cells. Valuable information on the metabolic processes will explain the response of cells.

### Metabolomic study

The metabolome perturbations were studied after exposing HaCaT cells to concentrations corresponding to IC_20_ and IC_50_ values: 0.10 mM and 0.30 mM for DCBQ-OH and 0.05 mM and 0.075 mM for DCBQ, respectively. Representative ^1^H-NMR spectra of the metabolomes from control and exposed cells can be seen in Fig. S10 and Fig. S11. Both concentrations of DCBQ-OH and DCBQ used in the metabolomic study are higher than those found in real-life aqueous samples. Although the concentration levels of the chemical factors employed in this study are not environmentally relevant, it is highly conceivable that they represent a pessimistic exposure scenario while the use of concentrations likely higher than those considered environmentally important are common among the (eco)toxicological assessments. Higher concentrations were unsuitable for the metabolomic study due to the high cell mortality, which means that many pathways would be deactivated and cells would malfunction.

### ^1^Η NMR metabolic fingerprinting

The HMDB spectral analysis yielded several metabolites, which were further verified by their mass spectra (all spectroscopic data for the identification of the metabolites are given in Table S1). A total of 98 metabolites were obtained for the cells exposed to both chemical factors. The metabolites identified in each sample are given in detail in Tables S2 and S3. When exposed to DCBQ-OH and DCBQ, 66 and 88 metabolites were obtained, respectively. Certain metabolites were common to cells exposed to both chemical factors. These include organic molecules belonging to organic acids, organoheterocyclic compounds, organic oxygen-containing, and nitrogen-containing compounds, fatty acyls, glycerophospholipids, sphingolipids, steroids, nucleosides, and nucleotides (Supporting Information-Metabolites).

### Metabolic pathway analysis

Pathway analysis took place to transform the results into biological information using the MetaboAnalyst 5.0 tool suite (“pathway analysis”). The metabolites identified in cells not exposed to the chemical factors were assigned to twenty-one different metabolic pathways (Table 1). In the case of DCBQ-OH, seventeen metabolic pathways were unaffected, as seen in Table 1 while other metabolic pathways were activated or blocked although being active in the control cells. The activation of them signifies the attempt of cells to adapt themselves to their environment and retain survivability. Exposure to the IC_20_ of DCBQ-OH yielded twenty-six metabolic pathways associated with the identified metabolites (Table 1). The following five metabolic pathways were activated: ascorbate and aldarate metabolism, biotin metabolism, cysteine and methionine metabolism, glutathione metabolism, and pyrimidine metabolism. Exposure of cells to the IC_50_ of DCBQ-OH resulted in twenty metabolic pathways (Table 1). The fructose and mannose metabolism, glycolysis/gluconeogenesis, lysine degradation, starch, and sucrose metabolism that appeared in the non-exposed cells and those exposed to the concentration of IC_20_ were downregulated in those exposed to IC_50_. Also, the metabolic pathways of α-linolenic acid metabolism, the biosynthesis of unsaturated fatty acids and cysteine and methionine metabolism were activated.

**Table 1:** Metabolic pathways of control HaCaT cells and treated with 0.10 mM and 0.30 mM of DCBQ-OH. Active metabolic pathways in the sample are denoted as + while inactive pathways are denoted as -

Metabolic Pathway	Control	IC_20_ (0.10 mM)	IC_50_ (0.30 mM)
Alanine, aspartate and glutamate metabolism	+	+	+
Amino sugar and nucleotide sugar metabolism	+	+	+
Aminoacyl-tRNA biosynthesis	+	+	+
Arachidonic acid metabolism	+	+	+
Arginine and proline metabolism	+	+	+
Arginine biosynthesis	+	+	+
D-Glutamine and D-glutamate metabolism	+	+	+
Folate biosynthesis	+	+	+
Galactose metabolism	+	+	+
Glycerolipid metabolism	+	+	+
One carbon pool by folate	+	+	+
Pentose and glucuronate interconversions	+	+	+
Primary bile acid biosynthesis	+	+	+
Sphingolipid metabolism	+	+	+
Steroid biosynthesis	+	+	+
Steroid hormone biosynthesis	+	+	+
Taurine and hypotaurine metabolism	+	+	+
Cysteine and methionine metabolism	-	+	+
Fructose and mannose metabolism	+	+	-
Glycolysis / Gluconeogenesis	+	+	-
Lysine degradation	+	+	-
Starch and sucrose metabolism	+	+	-
Ascorbate and aldarate metabolism	-	+	-
Biotin metabolism	-	+	-
Glutathione metabolism	-	+	-
Pyrimidine metabolism	-	+	-
α-Linolenic acid metabolism	-	-	+
Biosynthesis of unsaturated fatty acids	-	-	+

In the case of DCBQ, the metabolites arising from non-exposed cells were assigned to twenty-two metabolic pathways (Table 2). Preliminary experiments revealed that the presence of methanol did not cause any perturbations to metabolic pathways. Thus, the observed differences are ascribed, solely, to the DCBQ exposure. As mentioned above, exposure of the cells to DCBQ for 30 min has partially converted it into DCBQ-OH, the concentration of which does not affect the viability, as demonstrated by the 30-min exposure of cells to DCBQ-OH concentrations of 0.10 and 0.30 mM. Considering that (i) the concentrations of the DCBQ-OH formed are even lower than 0.10 mM where metabolic perturbations were observed earlier, and (ii) metabolic alterations differ from those found when cells were exposed to DCBQ, it is quite likely that metabolic alterations after exposure to DCBQ are attributed only to the parent compound.

Nineteen metabolic pathways were not affected by the DCBQ and they were present in all samples. Cell exposure to IC_20_ of DCBQ yielded metabolites assigned to thirty-one metabolic pathways (Table 2). The pathways of biotin metabolism and valine, leucine and isoleucine biosynthesis appearing in the non-exposed cells were downregulated at both concentrations. Eleven metabolic pathways were activated in samples exposed to IC_20_ of DCBQ, as seen below (Table 2). The cell exposure to IC_50_ of DCBQ yielded several metabolites assigned to thirty metabolic pathways (Table 2). Even though lysine degradation appeared in the non-exposed cells and those exposed to IC_20_, in IC_50_ samples this was downregulated. The following seven metabolic pathways, not encountered in the control sample, were activated when cells were exposed to DCBQ, at both concentrations: arginine biosynthesis, D-glutamine, and D-glutamate metabolism, fatty acid degradation, glycerophospholipid metabolism, lipoic acid metabolism, the pathway of one carbon pool by folate and riboflavin metabolism. The following four new metabolic pathways not occurring in the control and exposed to IC_20_ samples were activated in IC_50_ samples: glycine, serine, and threonine metabolism, inositol phosphate metabolism, pentose phosphate pathway, and phosphatidylinositol signaling system (Table 2). It is reasonable that the metabolic pathways of the control samples employed for DCBQ-OH and DCBQ, exhibit distinct differences because the cells for the study of DCBQ were incubated for 30 min in DMEM of pH = 5.0. The differences in the metabolic pathways mentioned above will further be discussed below.

**Table 2:** Metabolic pathways of control HaCaT cells and treated with 0.05 mM and 0.075 mM of DCBQ. Active metabolic pathways in the sample are denoted as +, while inactive pathways are denoted as -

Metabolic Pathway	Control	IC_20_ (0.05 mM)	IC_50_ (0.075 mM)
Alanine, aspartate and glutamate metabolism	+	+	+
Amino sugar and nucleotide sugar metabolism	+	+	+
Aminoacyl-tRNA biosynthesis	+	+	+
Arachidonic acid metabolism	+	+	+
Arginine and proline metabolism	+	+	+
Ascorbate and aldarate metabolism	+	+	+
Fructose and mannose metabolism	+	+	+
Galactose metabolism	+	+	+
Glycerolipid metabolism	+	+	+
Glycolysis / Gluconeogenesis	+	+	+
Neomycin, kanamycin and gentamicin biosynthesis	+	+	+
Pentose and glucuronate interconversions	+	+	+
Primary bile acid biosynthesis	+	+	+
Pyrimidine metabolism	+	+	+
Sphingolipid metabolism	+	+	+
Starch and sucrose metabolism	+	+	+
Steroid biosynthesis	+	+	+
Steroid hormone biosynthesis	+	+	+
Taurine and hypotaurine metabolism	+	+	+
Lysine degradation	+	+	-
Arginine biosynthesis	-	+	+
D-Glutamine and D-glutamate metabolism	-	+	+
Fatty acid degradation	-	+	+
Glycerophospholipid metabolism	-	+	+
Lipoic acid metabolism	-	+	+
One carbon pool by folate	-	+	+
Riboflavin metabolism	-	+	+
Biotin metabolism	+	-	-
Valine, leucine and isoleucine biosynthesis	+	-	-
β-Alanine metabolism	-	+	-
Cysteine and methionine metabolism	-	+	-
Glutathione metabolism	-	+	-
Histidine metabolism	-	+	-
Pentose phosphate pathway	-	-	+
Phosphatidylinositol signaling system	-	-	+
Glycine, serine and threonine metabolism	-	-	+
Inositol phosphate metabolism	-	-	+

### Relative quantification of metabolites

**Fig. 1:**
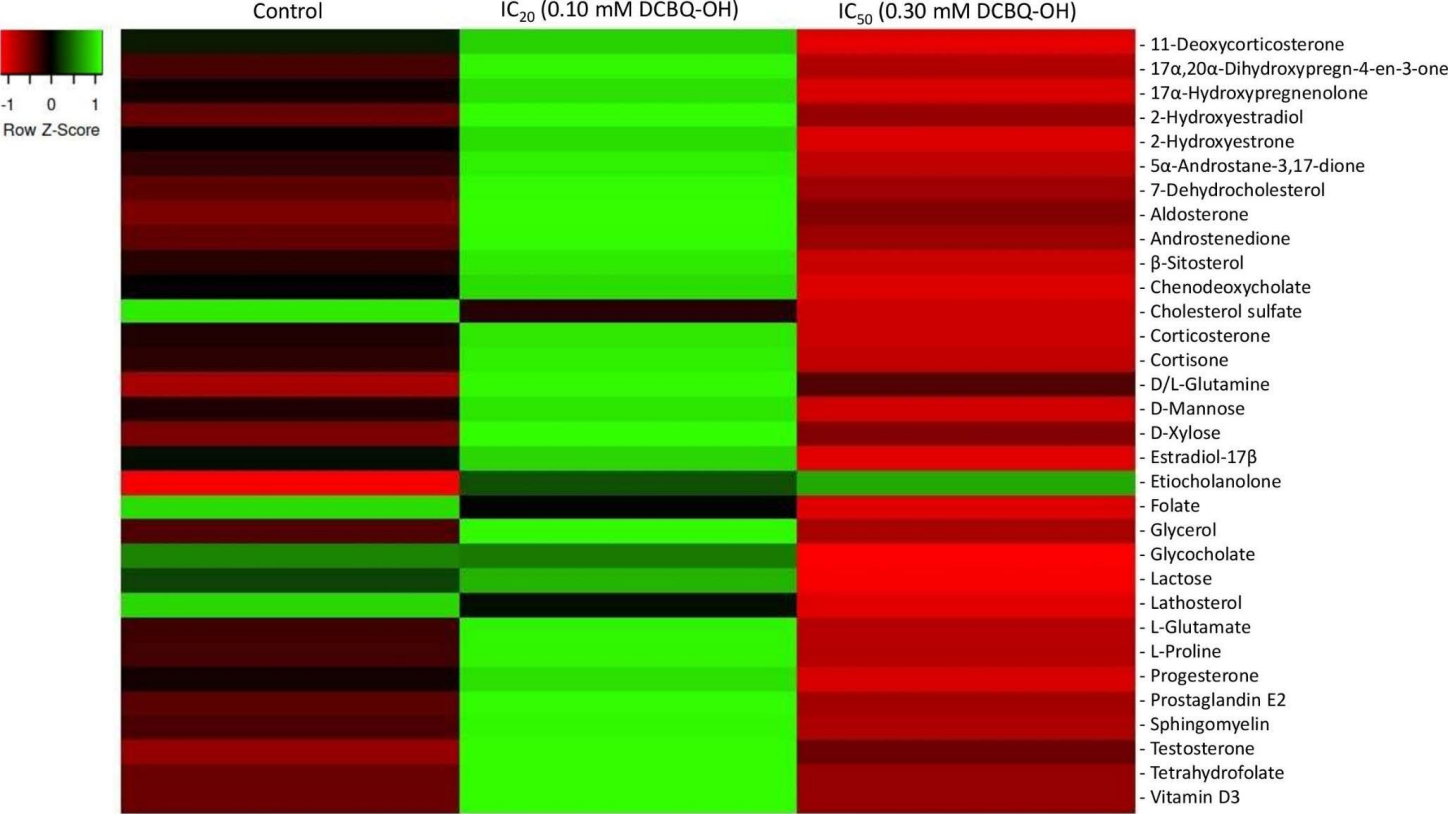
Heatmap with the metabolites of control HaCaT and treated with 0.10 mM and 0.30 mM of DCBQ-OH.

All data regarding the relative quantification of the metabolites are given in detail in Tables S4 and S5. Thirty-two of the metabolites were found to be present in control cells and cells after exposure to IC_20_ and IC_50_ of DCBQ-OH. The heatmap (Fig. 1) shows the quantitative alteration of significant metabolites after exposure to DCBQ-OH. The relative content of most of these metabolites increased when the cells were exposed to the low concentration of DCBQ-OH (i.e., IC_20_) and decreased at the high concentration (i.e., IC_50_), as compared with the control. When exposed to the IC_20_ of DCBQ-OH, a significant increase in the relative content was observed for the 2-hydroxyestradiol, 7-dehydrocholesterol, androstenedione, D-xylose, and the L-glutamate, reaching percentages of 125%, 145%, 283%, 215%, and 256%, respectively. After exposure to IC_50_ of DCBQ-OH, a major decrease in the relative content was noticed for β-sitosterol and D-mannose, reaching a decrease of 62% in both. The relative content of cholesterol sulfate, folate, glycocholate, and lathosterol appeared to decrease in a dose-dependent manner when cells were exposed to DCBQ-OH. For cholesterol sulfate, the decrease was 42% and 70% at IC_20_ and IC_50_, respectively, while for folate the respective decrease was 40% and 82%. On the other hand, the relative content of etiocholanone appeared to increase by 32% and 41%, at the low and high concentrations, respectively.

**Fig. 2:**
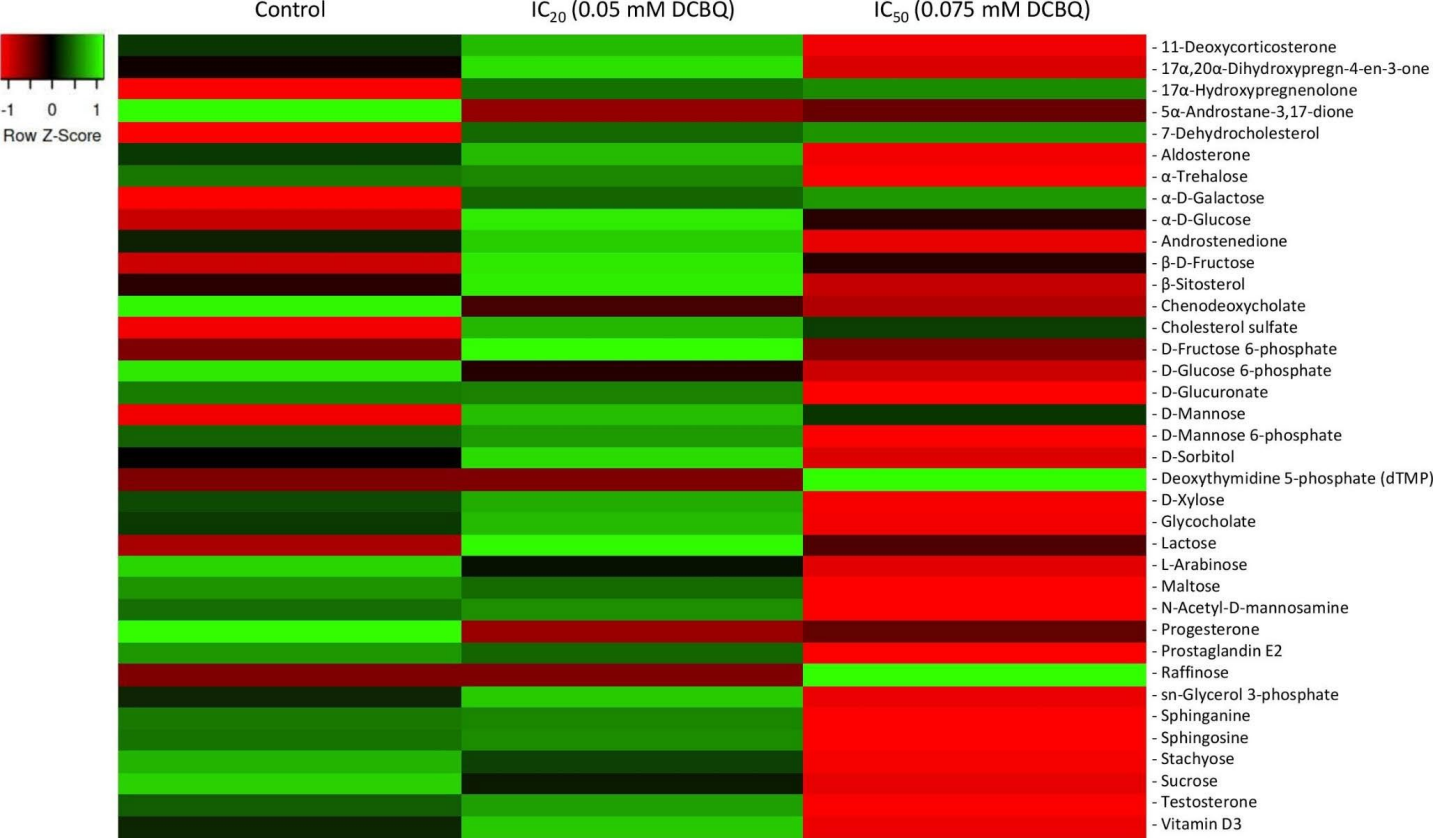
Heatmap with the metabolites of control HaCaT and treated with 0.05 mM and 0.075 mM of DCBQ.

In the case of DCBQ, thirty-seven metabolites were identified in both control and exposed (IC_20_ and IC_50_) cell samples. Most cellular metabolites exhibited an increase in their relative content at the low concentration of DCBQ (i.e., IC_20_) and a decrease when cells were treated with the high one (i.e., IC_50_), as seen in the heatmap of Fig. 2. Specifically, β-sitosterol exhibited an increase of 38% and a decrease of 21%, D-xylose exhibited an increase of 19% and a decrease of 65%, and vitamin D3 exhibited an increase of 37% and a decrease of 61%. The relative content of 17α-hydroxypregnenolone, α-D-glucose, and β-D-fructose increased as DCBQ increased. The relative contents of 5α-androstane-3,17-dione, chenodeoxycholate, D-glucose 6-phosphate, L-arabinose, maltose, progesterone, prostaglandin E2, stachyose, and sucrose in cell samples significantly diminished, as the DCBQ concentration increased. The chenodeoxycholate exhibited a decrease of 40% and 54% and the relative content of prostaglandin E2 decreased by 8% and 64%, at the IC_20_ and IC_50_, respectively. The respective decrease in stachyose was 26% until its NMR peak was not possible to be integrated. In sucrose the corresponding decrease was 30% and 71%.

## Discussion

The DCBQ and its hydroxyl derivative DCBQ-OH belong to a class of DBPs that are of toxicological relevance and carcinogenic potency. The spontaneously immortalized human keratinocyte cell line HaCaT from adult skin is an ideal model for the study of keratinocyte functions and responses upon stimulation. This study represents the first attempt to study the metabolism of HaCaT cells after exposure to the two DBPs.

Prior work investigating the two DBPs reported that IC_50_ values of DCBQ and DCBQ-OH in Chinese hamster ovary cells (CHO-K1) after 24 h of exposure were 27.3 µM and 61.0 µM, respectively (Wang et al., 2014). For HEK-V kidney (Li et al., 2017) and HepG2 cells (Wang et al., 2018) the IC_50_ of DCBQ for a 24 h exposure period was found to be 44.0 µM and 72.0 µM, respectively. Herein, from the cell viability study, it was deduced that the IC_50_ values of DCBQ and DCBQ-OH after exposure to them of the more resilient HaCaT cells were 0.075 mM and 0.30 mM, respectively.

In the case of cells, exposed to the low concentration of DCBQ-OH (equal to IC_20_), certain metabolic pathways were activated. For instance, the activation of ascorbate and aldarate metabolism, glutathione, methionine and cysteine, and pyrimidine metabolism indicates that the exposure of cells induces oxidative stress. Specifically, ascorbate and aldarate, as antioxidants consume oxygen-free radicals and contribute to the preservation of α-tocopherol, an important antioxidant of the cell membrane (May, 1998). Glutathione protects cell membranes from free radicals and reactive oxygen intermediates (Meister, 1983) while homocysteine, a metabolite that takes part in the cysteine and methionine metabolism, can act as a precursor in the synthesis of glutathione (Portillo et al., 2020). Finally, if mitochondria DNA is exposed to reactive oxygen species pyrimidines play a repairing role in it, if damage occurs (Garavito et al., 2015). In addition, exposure to DCBQ-OH can lead to a greater demand of cells for energy. This is evident in the case of activation of biotin metabolism, which provides cells with energy. Biotin can act as a coenzyme of carboxylases involved in the normal metabolism of proteins, lipids, and carbohydrates. Also, it is necessary for the biosynthesis of fatty acids, the catabolism of branched-chain amino acids, and odd-chain fatty acids; besides it takes part in gluconeogenesis (Pacheco-Alvarez et al., 2002).

The α-linolenic acid and unsaturated fatty acids are structural components of cell membranes and affect the flexibility and permeability of membranes and enzyme activity. The activation of the above pathways after exposure of cells to the high concentration of DCBQ-OH (equal to IC_50_) underlines the perturbation of cells. Both pathways along with the activation of cysteine and methionine metabolism at the concentration of IC_50_ suggest induced oxidative stress in cells. The deactivation of certain pathways appearing in both control and exposed to DCBQ-OH IC_20_ samples, such as fructose, mannose, starch and sucrose metabolism, glycolysis/gluconeogenesis, and lysine degradation indicate that IC_50_ affected cells adversely by hindering energy production. Fructose, mannose, starch, and sucrose are saccharides and their metabolism provides cells with glucose. Lysine degradation yields two acetyl coenzymes A, which can be oxidized for energy production (Leandro & Houten, 2020).

In living organisms, reactive oxygen species are generated as a product of normal metabolism (Nita & Grzybowski, 2016). The DCBQ-OH at concentrations of 0.10 mM and 0.30 mM had 82% and 95% scavenging activity on DPPH radical. These figures are in contrast to our assertions that DCBQ-OH forms reactive species. In a previous study, Hung et al. demonstrated that the water-transformed DCBQ led to the formation of intracellular reactive oxygen species, based on the 2,7-dichlorofluorescin diacetate assay, thus acting as a pro-oxidant (Hung et al., 2019). In our case, the treatment of HaCaT cells with DCBQ-OH brought out its role as a pro-oxidant rather than as an antioxidant, based on the activated metabolic pathways, indicating that oxidative stress occurred.

The relative quantification of the metabolites in the samples exposed to DCBQ-OH and the control samples evidenced a disturbance of membranes. The relative content of membrane components, such as glycerol, sphingomyelin, and L-proline, increased at IC_20_ in order to protect the membrane. This content decreased rapidly after exposure to IC_50_ because of the induced perturbation by the DCBQ-OH, which was higher at the high concentration. These metabolites contribute to the enhancement and protection of cellular membranes. Moreover, lathosterol content decreased by 46% and reached non-detectable levels at IC_20_ and IC_50_ of DCBQ-OH, respectively, leading to the production of cholesterol. Under the above conditions, cholesterol sulfate decreased by 42% and 70%, respectively, in favor of the production of steroids and steroid hormones for enhancing membranes.

In the case of exposure of cells to DCBQ, the differences in metabolic pathways were attributed, mainly, to DCBQ, considering that DCBQ-OH was present at so low concentrations that they did not affect the metabolism. When the cells were exposed to the IC_20_ and IC_50_ of DCBQ, the biotin metabolism and valine, leucine, and isoleucine biosynthesis were downregulated. As mentioned above, biotin is involved in gluconeogenesis, while isoleucine plays a prominent role in enhancing glucose consumption and utilization (Zhang et al., 2017). It is evident that DCBQ prevents cells from making use of glucose. In addition, when cells were exposed to the IC_50_ of DCBQ, lysine degradation was downregulated contributing to energy production. As a result, the cells activated other pathways to satisfy their energy demands. Riboflavin metabolism was activated when cells were exposed to both concentrations. Riboflavin is a water-soluble micronutrient that supports energy production by assisting in the metabolism of fats, carbohydrates, and proteins (Powers, 2012). The pentose phosphate pathway was activated when cells were exposed to the IC_50_ of DCBQ. This pathway does not provide cells with ATP to meet their energy demands; instead, it yields NADPH and ribose-5-phosphate, which are vital for their survival (Ge et al., 2020).

Furthermore, when cells were exposed to both low or high concentrations, perturbations on the cell membranes occurred as arginine biosynthesis, glycerophospholipid metabolism, and fatty acid degradation were activated. Through arginine biosynthesis, ornithine is produced, which leads to the production of collagen (Tong & Barbul, 2004). Glycerophospholipids are components of cell membranes (Hermansson et al., 2011) while fatty acid degradation is attributed to cell death and degraded components of membranes of the living cells. The DCBQ induced oxidative stress on cells as D-glutamine and D-glutamate, lipoic acid metabolism, and the pathway of one carbon pool by folate were activated when exposed to IC_20_ and IC_50_ of DCBQ. D-Glutamine, D-glutamate, and folate are precursors of glutathione and lipoic acid is a vitamin-like antioxidant that behaves like a free radical scavenger.

Other pathways which support oxidative stress, are β-alanine, cysteine, methionine, glutathione, and histidine metabolism, which were activated only at the low concentration of DCBQ. β-Αlanine and histidine are precursors of carnosine, an antioxidant and free-radical scavenging factor (Vraneš et al., 2021), while cysteine and methionine are precursors of glutathione, as mentioned above. Cells exposed to IC_50_ of DCBQ activated glycine, serine and threonine metabolism as a way of protecting themselves from oxidative stress and membrane damage. Glycine is a precursor of glutathione (Wang et al., 2013a, b) and serine acts as an antioxidant (Naderi Maralani et al., 2012). Both glycine and threonine are components of collagen, a key ingredient of cell membranes. Finally, cells exposed to IC_50_ of DCBQ activated inositol phosphate metabolism and phosphatidylinositol signaling system. Inositol phosphates are important components of lipids, known as phosphatidylinositols, which are key components of cell membranes. These two pathways, also, were involved in the transport of messages from the receptors of cell membranes to the interior of cells (Berridge, 2009). The phosphorylated derivatives of inositol act as second messengers in signal transduction pathways for the adaptation to environmental stress and intercellular communication. The messages transferred involve the control of functions, such as secretion, metabolism, and growth (Berridge & Irvine, 1989).

The IC_20_ and IC_50_ of DCBQ exhibited a DPPH radical scavenging activity of 50% and 64%, respectively. These findings are in contradiction to our expectations that DCBQ can form reactive species. In previous studies, DCBQ exhibited pro-oxidant activity (Du et al., 2013; Sun et al., 2019). Specifically, the 2,7-dichlorofluorescin diacetate assay showed that the production of reactive oxygen species increased when the concentration of DCBQ increased (Du et al., 2013) and malondialdehyde production increased as DCBQ increased (Sun et al., 2019). From the activated pathways, it is evident that DCBQ acts more as a pro-oxidant in in vitro cell experiments than as an antioxidant, just like DCBQ-OH.

From the relative content of the metabolites present in the samples exposed to DCBQ, some findings deserve further discussion. The relative content of α-D-glucose increased reaching percentages of 295% and 110% compared with the control samples. Pathways involved in glucose utilization like biotin metabolism and valine, leucine, and isoleucine biosynthesis were downregulated, signifying that glucose was accumulated. Also, α-trehalose was activated, when cells were exposed to DCBQ. It is worth mentioning that this saccharide, not found in cells exposed to DCBQ-OH, prevents cells from dehydrating and disrupting their internal organelles. Exposure of cells to the low concentration of DCBQ barely increased its relative content. By contrast, when the cells were exposed to the high concentration, all the content of saccharides was consumed following the patterns of maltose, stachyose, and sucrose, which decreased as DCBQ increased. All these saccharides broke down to give glucose, as the energy demands of cells were rapidly increasing. The 7-dehydrocholesterol, a steroid component of cell membranes, increases as cells produce it to enhance and protect their membranes. Sphinganine and sphingosine are components of cell membranes and their content increased when exposed to the low concentration and decreased rapidly when exposed to the high concentration, where the perturbation of cell membranes was greater. It is evident that DCBQ disrupts cells, to a considerable degree.

## Conclusion

This study examined the effect of DCBQ and its hydroxyl analogue DCBQ-OH on the metabolism of HaCaT. Both compounds were toxic to HaCaT cells indicating their negative effects on humans, after exposure. Each of the two chemical factors was studied at two different concentrations, corresponding to the IC_20_ and IC_50_ , in order to obtain valuable information on cell metabolome. Both of them brought about multiple metabolomic alterations in the HaCaT cells with them being more vulnerable to DCBQ than its hydroxyl analogue. Certain metabolic pathways were downregulated whereas others were activated to assist in their survivability. As DCBQ is more cytotoxic than DCBQ-OH and brought about more alterations in the metabolic pathways it is safe to conclude that it may have a great impact on living cells. The knowledge of the key molecules that can trigger the diverse metabolic pathways in cells exposed to the above chemical factors may allow us to know more about the regulatory mechanisms involved in metabolite generation. However, further studies would still be needed to elucidate further the implications of such exposures and bridge the remaining knowledge gaps in this area.

## Electronic supplementary material

Below is the link to the electronic supplementary material.

Supplementary Material 1

## Data Availability

Data are available by the corresponding author, upon request.
