# Supplementary material for: Untargeted metabolomics of human keratinocytes reveals the impact of exposure to 2,6-dichloro-1,4-benzoquinone and 2,6-dichloro-3-hydroxy-1,4-benzoquinone as emerging disinfection by-products
Source: Metabolomics. 2022 Nov 7;18(11):89. doi: 10.1007/s11306-022-01935-2 (PMC9640400; doi:10.1007/s11306-022-01935-2)
Supplement: Supplementary file 1 — Supplementary Material 1 [file 11306_2022_1935_MOESM1_ESM.docx]

**ELECTRONIC SUPPLEMENTARY MATERIAL**

**Untargeted metabolomics of human keratinocytes reveals the impact of exposure to 2,6-dichloro-1,4-benzoquinone and 2,6-dichloro-3-hydroxy-1,4-benzoquinone as emerging disinfection by-products**

Dimitra G. Meintani^a^, Theodoros G. Chatzimitakos^a^, Athanasia I. Kasouni^b^ and Constantine D. Stalikas^a,*^

^a^ Laboratory of Analytical Chemistry, Department of Chemistry, University of Ioannina, 45110 Ioannina, Greece

^b^ Laboratory of Biophysical Chemistry, Department of Biological Applications and Technologies, University of Ioannina, 45110 Ioannina, Greece

* Corresponding author. e-mail: cstalika@uoi.gr, Fax: ××30 26510 08796

**1. Instrumentation**

^1^H-NMR spectra were recorded on a Brüker AV-500 spectrometer equipped with a TXI cryoprobe (Bruker BioSpin, Rheinstetten, Germany). The NMR system was controlled by the software TopSpin 4.0.7. (Copyright 2009, Bruker BioSpin). All spectra were acquired with an acquisition time of 2.339 s, relaxation delay of 6.50 μs, 32 K data points, 30° pulse length, at temperature 298 K and 512 scans. Phase and baseline were manually corrected after the Fourier transform.

Metabolites were further analyzed using an Ultimate 3000 HPLC (Dionex, Milan, Italy) ultra-high-performance liquid chromatographic (UHPLC) system. The chromatographic column Hypersil GOLD 1.9 μm particle size (100 mm × 2.1 mm I.D), was used. Oven temperature remained at 30^o^C. The mobile phase used consisted of water (A) and acetonitrile (B) (formic acid 0.1% v/v). For the separation of metabolites a gradient program was employed: 0-13.78 min, 20-90% B, 13.78-15.28 min, 90% B, 15.28-18.06 min, 90-20% B, followed by a 2-min re-equilibration time of the column. The flow rate of the mobile phase was 300.0 μL min^-1^.

Afterwards, metabolites were detected using a linear trap quadrupole (LTQ) Orbitrap mass spectrometer (Thermo Scientific, Bremen, Germany), equipped with an atmospheric pressure interface and an ESI ion source. Effluents from the chromatographic system were delivered to the ion source with nitrogen as the sheath and auxiliary gas. Ionization was carried out both in positive and negative modes. Sample injection volume was 2.5 μL, source voltage was 3.4 kV, tube lens was 110 V, while the heated capillary voltage was 40.0 V and temperature was maintained at 320 °C, for the positive ionization mode. Sample was injected in a volume of 10.0 μL, source voltage was 3.7 kV, tube lens was 120 V, the heated capillary voltage was -30.00 V and temperature was maintained at 320 °C, for the negative ionization. A full scan mode, at a resolution of 60,000 and an m/z range of 50-1500, was used. Then a most-intense-ion scan (MS/MS fragmentation of the most abundant ion), with a resolution of 7,500 was used. The system was controlled by the Thermo Xcalibur 2.5.0 software.

A UV-Vis Shimadzu-2100 Spectrophotometer of spectra (Shimadzu) was used for the acquisition of spectra. Forma direct heat CO_2_ incubator (Thermo Electron Corporation, America), EUROCLONE Fume Hood (EUROCLONE, Italy), and microscope Olympus BX43 (Lumenera, Canada) were used for the experiments on cells.

**2. Cell viability assay**

When evaluating the cell viability on exposure to DCBQ, the pH of DMEM was adjusted to 5.0 and the exposure of cells was performed in darkness, to lower the conversion rate of DCBQ to DCBQ-OH (Hung et al., 2019). The volume of methanol used in the exposure of cells to DCBQ did not exceed 10.0 μL, to avoid cell perturbation from methanol. Control samples without any DBP were prepared in DMEM with the addition of DDW and methanol, as these were the solvents of DCBQ-OH and DCBQ, respectively. Samples with DMEM pH=7.6 and control samples with DMEM pH=5.0 were used to evaluate whether the change of pH affected the viability of cells. DMEM was discarded and cells were washed with PBS. Crystal violet assay was performed to measure the cell viability (Chatzimitakos et al., 2018). The experiments were carried out in triplicate. The loss of viability was expressed as a % percentage of the control sample.

**Fig. S1:** Molecular absorption spectra of DCBQ-OH 1.0 mM after exposure to daylight for 24, 48, 72, and 96 h.

**
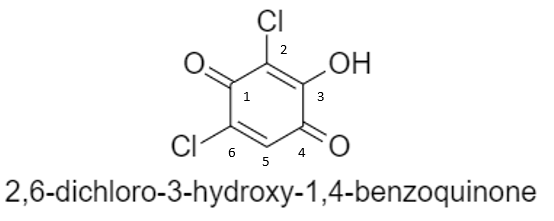

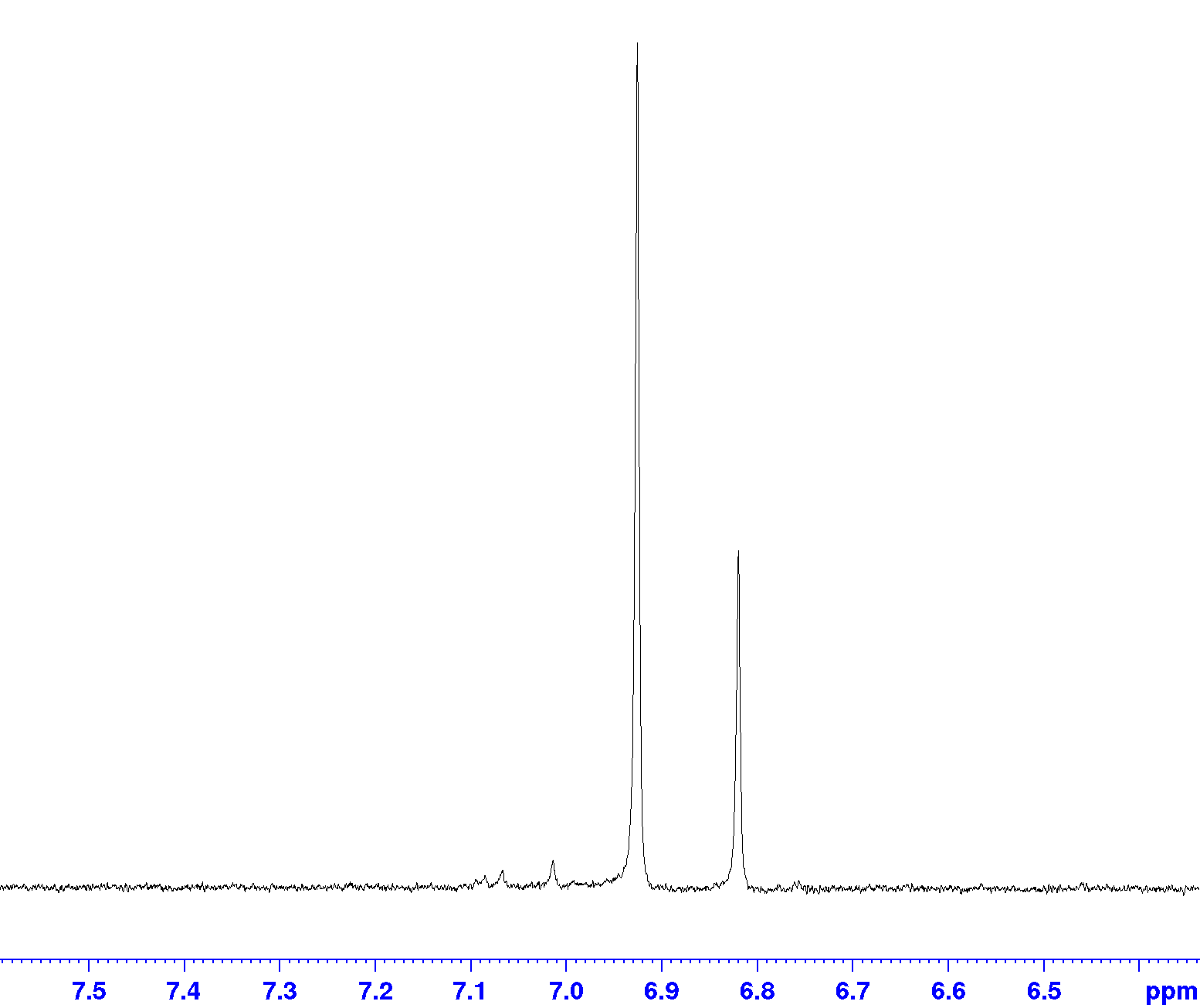
**

H_OH_

H_C5_

**
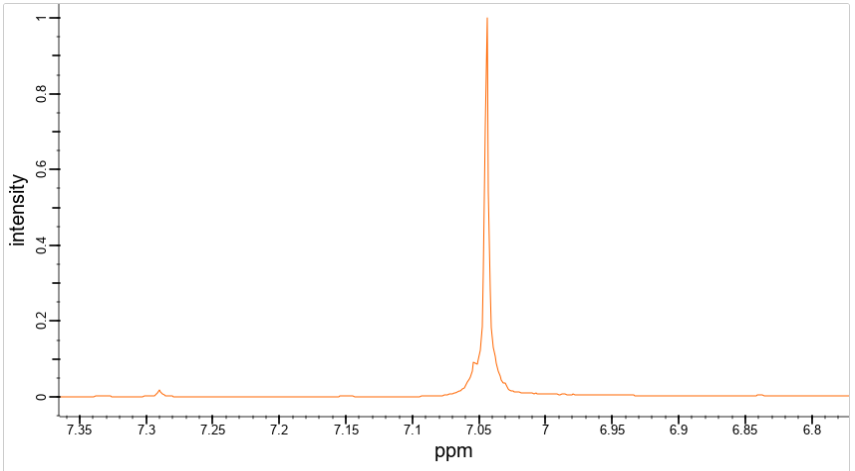
Fig. S2:** ^1^H-NMR spectrum of DCBQ-OH 1.0 mM.

**
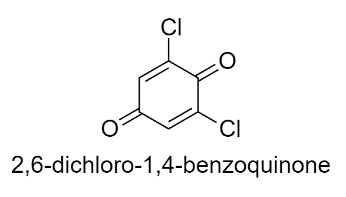
**

**Fig. S3:** ^1^H-NMR spectrum of DCBQ (*https://spectrabase.com/spectrum/sDuzxU6xI* (Wiley & Sons, 2021)).

**Fig. S4:** Kinetics of DCBQ-OH, at 530 nm, formed from DCBQ in DMEM pH=4.5, 5.5, 6.5, and 7.6.

**Fig. S5:** Kinetics of DCBQ-OH, at 530 nm, formed from DCBQ 0.05 mM and 0.075 mM, in DMEM pH=5.0 (experiments done in triplicate).

*

*

*

*

*

**Fig. S6:** DPPH scavenging activity (%) of DCBQ-OH (orange bar) and DCBQ (blue bar) (experiments done in triplicate. *Statistically significant difference p<0.05).


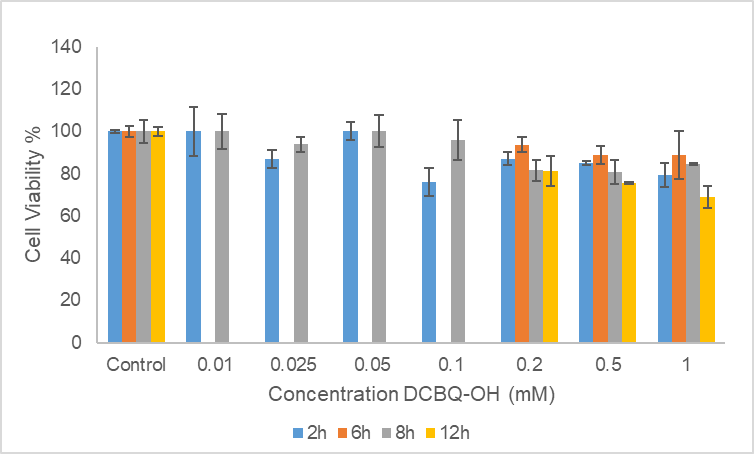


*

*

*

*

**Fig. S7:** Viability (%) of HaCaT cells exposed to 0.01-1.0 mM DCBQ-OH for 2, 6, 8 and 12 h (experiments done in triplicate. *Statistically significant difference p<0.05).

*

*

*

**Fig. S8:** Viability (%) of HaCaT cells exposed to 0.10-1.25 mM DCBQ-OH for 24 h. At 1.25 mM DCBQ-OH all cells have died (experiments done in triplicate. *Statistically significant difference p<0.05).

*

*

*

*

**Fig. S9:** Viability (%) of HaCaT cells exposed to 0.01-0.30 mM DCBQ for 30 min. At 0.3 mM DCBQ all cells have died (experiments done in triplicate. *Statistically significant difference p<0.05).


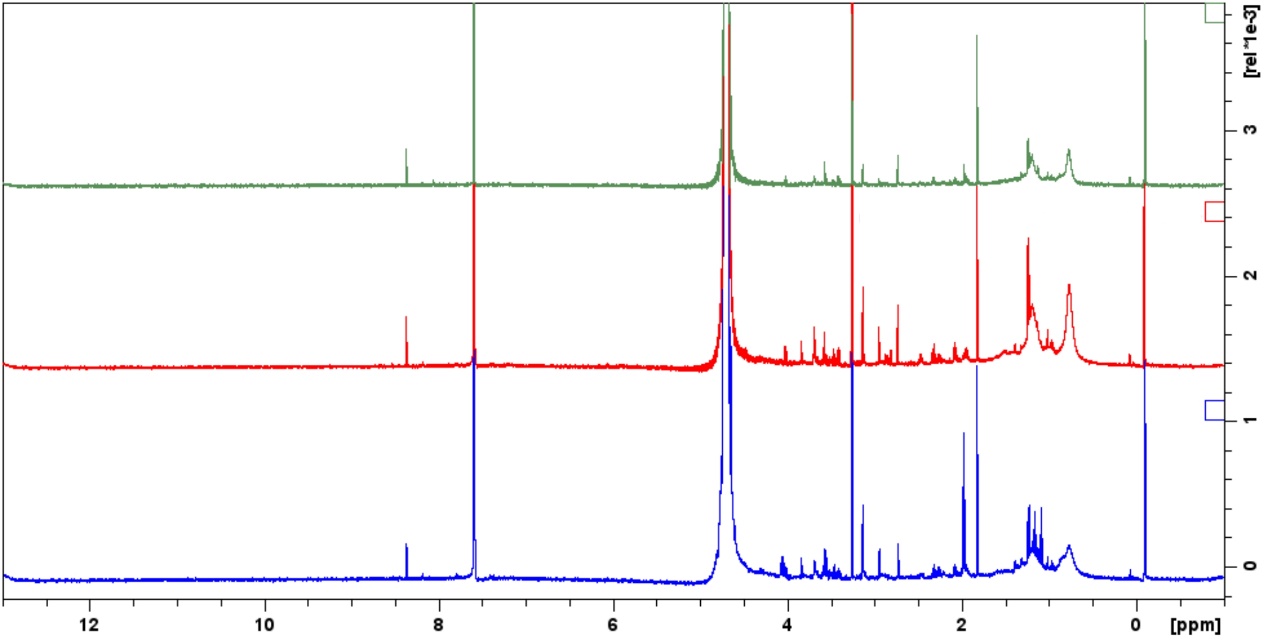


**Fig. S10**: Representative ^1^H-NMR spectra of the metabolomes of control HaCaT cells (blue spectrum) and cells exposed to 0.10 mM (red spectrum) and 0.30 mM (green spectrum) of DCBQ-OH.


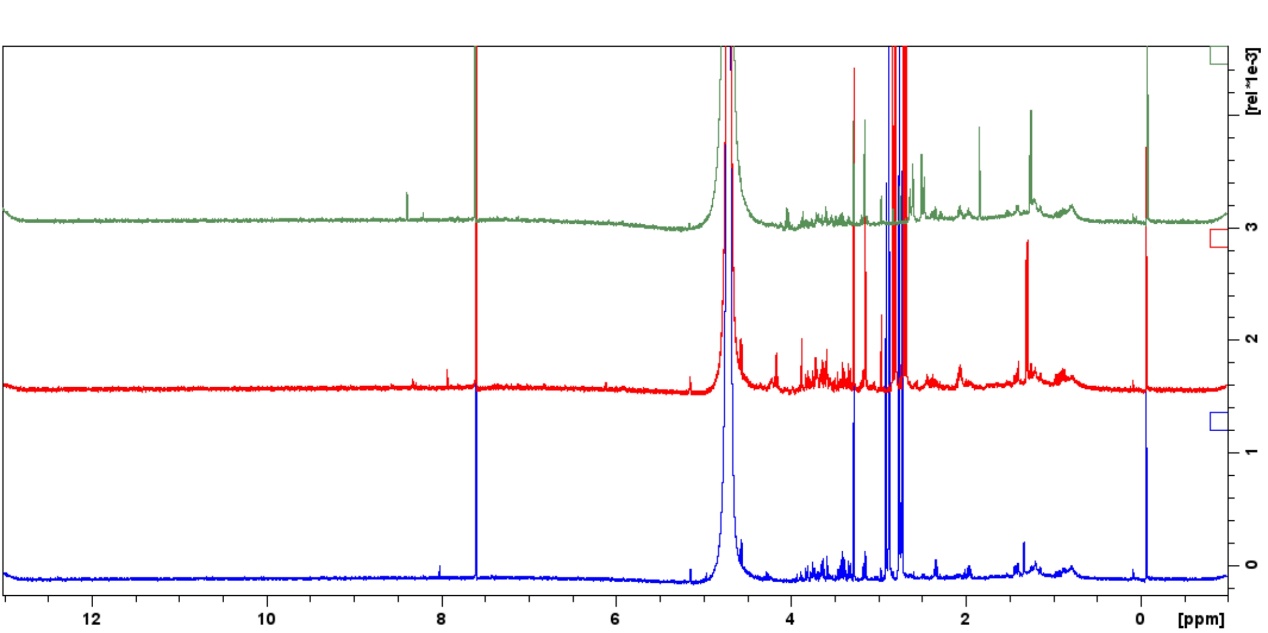


**Fig. S11**: Representative ^1^H-NMR spectra of the metabolomes of control HaCaT cells (blue spectrum) and cells exposed to 0.05 mM (red spectrum) and 0.075 mM (green spectrum) of DCBQ.

**Table S1**: Metabolite names, chemical formulas, CAS numbers and spectroscopic data employed for the identification of the metabolites.

| **Metabolite** | **Chemical formula** | **CAS Number** | **MS/MS fragmentation** | **^1^H-NMR characteristic signals** |
| --- | --- | --- | --- | --- |
| (9Z,12Z,15Z)-Octadecatrienoic acid | C_18_H_30_O_2_ | 463-40-1 | 219.2107/177.1637/  163.1481 /123.1168 | (2.030-2.110 m) |
| (R)-Lipoate | C_8_H_14_O_2_S_2_ | 1200-22-2 | 155.0525/129.0732/  113.0419 | (2.390 t. 7.410 Hz) |
| 11-Deoxycorticosterone | C_21_H_30_O_3_ | 64-85-7 | 109.1730/97.2260 | (1,190 s), (1,450 dd, 12,630/3,430 Hz), (1,580 dd, 11,230/3,320 Hz) |
| 17alpha,20alpha-Dihydroxypregn-4-en-3-one | C_21_H_32_O_3_ | 652-69-7 | 297.2213/243.1743/39.0229 | (1.020 d. 6.270 Hz), (1.140 s), (3.510 s) |
| 17alpha-Hydroxypregnenolone | C_21_H_32_O_3_ | 387-79-1 | 168.0000/156.0000/91.0000 | (0.940 s), (1.070-1.180 m), (3.220-3.300 m) |
| 17alpha-Hydroxyprogesterone | C_21_H_30_O_3_ | 68-96-2 | 109.0430/97.0670/81.1030 | (1.120 s), (2.200 s), (2.220 s) |
| 2-Hydroxyestradiol | C_18_H_24_O_3_ | 362-05-0 | 176.7760/154.4350/91.9600 | (3.490 td) |
| 2-Hydroxyestrone | C_18_H_22_O_3_ | 362-06-1 | 206.6610/156.5970/102.746/79.1910 | (2.410 dd) |
| 2-Methoxy-17beta-estradiol | C_19_H_26_O_3_ | 362-07-2 | 152.0000/151.0000/  134.0000 | (3.480-3.550 m) |
| 2-Methoxyestrone | C_19_H_24_O_3_ | 362-08-3 | 137.0000/131.0000/  129.0000/103.0000 | (0.830 s), (1.430-1.510 m), (2.110-2.210 m) |
| 5alpha-Androstane-3,17-dione | C_19_H_28_O_2_ | 846-46-8 | 175.1117/165.1274/93.0699 | (1.030 s), (1.050 s), (1.290-1.320 m), (1.340 dd. 12.170/5.010 Hz). (1.370/1.380 d. 4.000 Hz), (2.300-2.360 m), (2.390 dd. 13.810/6.510 Hz) |
| 5-Oxo-D-proline | C_5_H_7_NO_3_ | 98-79-3 | 84.0750/56.1320/41.3370 | (2.340-2.440 m), (4.170 dd. 9.020/5.830 Hz) |
| 7-Dehydrocholesterol | C_27_H_44_O | 434-16-2 | 95.2010/81.1030/69.2300 | (0.960 s), (0.990-1.070 m), (2.060-2.140 m) |
| Aldosterone | C_21_H_28_O_5_ | 52-39-1 | 170.0701/146.0701 | (1.170 s). (1.210 s) |
| alpha-Trehalose | C_12_H_22_O_11_ | 99-20-7 | 325.1129/281.0867/181.070 /163.0600/119.0338 | (3.440 t. 9.470 Hz), (3.730-3.780 m) |
| alpha-D-Galactose | C_6_H_12_O_6_ | 3646-73-9 | 145.0495/103.0389/91.0389 | (3.480 dd. 9.890/7.880 Hz), (3.640 dd. 9.910/3.500 Hz), (3.920 d. 3.07 Hz). (3.980 d. 2.660 Hz), (4.070 t. 5.980 Hz) |
| alpha-D-Galactose 1-phosphate | C_6_H_13_O_9_P | 2255-14-3 | 98.9847/80.9742/59.0133 | (3.712-3.767 m) |
| alpha-D-Glucose | C_6_H_12_O_6_ | 492-62-6 | 163.0600/145.0495/  103.0389 /91.0389 | (3.230 dd. 9.410/7.980 Hz), (3.880 dd. 12.300/2.230 Hz), (5.220 d 3.800 Hz) |
| alpha-D-Glucose 1,6-bisphosphate | C_6_H_14_O_12_P_2_ | 10139-18-1 | 183.0058/143.0344/98.9847 | (4.047-4.105 m) |
| Androstenedione | C_19_H_26_O_2_ | 63-05-8 | 108.0000/97.0000/96.0000 | (1.230-1.340 m), (2.060-2.150 m) |
| beta-Alanyl-N(pi)-methyl-L-histidine | C_10_H_16_N_4_O_3_ | 584-85-0 | 178.5000/122.0713/44.0495 | (3.040 dd. 15.790/8.970 Hz), (3.780 s) |
| beta-D-Fructose | C_6_H_12_O_6_ | 53188-23-1 | 117.0000/85.0000/73.0000 | (3.538-3.622 m), (4.096-4.141 m) |
| beta-D-Fructose 2,6-bisphosphate | C_6_H_14_O_12_P_2_ | 79082-92-1 | 98.9847/73.0290 | (3.615-3.668 m) |
| beta-D-Glucose 6-phosphate | C_6_H_13_O_9_P | 15209-12-8 | 145.0501/103.0395/98.9847 | (3.490-3.595 m) |
| beta-Sitosterol | C_29_H_50_O | 83-46-5 | 95.0790/81.0670/57.1890 | (1.010 s), (3.480-3.580 m) |
| Biotin | C_10_H_16_N_2_O_3_S | 58-85-5 | 210.058/199.0899/157.0430 | (2.820 dd. 12.410/5.090 Hz), (4.080-4.180 m) |
| Chenodeoxycholate | C_24_H_40_O_4_ | 474-25-9 | 107.0000/95.0000/93.0000/  81.0000 | (0.914 s), (0.929 d. 6.560 Hz), (2.212 td. 9.410/5.030 Hz). (3.885 ddd) |
| Cholesterol ester (16:0) | C_43_H_76_O_2_ | 601-34-3 | 357.3521/329.3208/57.0704 | (0.940-1.020 m) |
| Cholesterol ester CE(12:0) | C_39_H_68_O_2_ | 1908-11-8 | 357.3521/329.3208/57.0704 | (0.908-0.957 m) |
| Cholesterol sulfate | C_27_H_46_O_4_S | 1256-86-6 | 451.2882/71.0861/57.0704/  55.0548 | (1.190-1.270 m), (2.090-2.170 m) |
| CoA | C_21_H_36_N_7_O_16_P_3_S | 85-61-0 | 708.1191/654.1506/  564.0656/261.1267/  136.0617 | (2.460 t. 6.550 Hz), (6.170 d. 7.040 Hz) |
| Corticosterone | C_21_H_30_O_4_ | 50-22-6 | 121.0090/105.0670/97.0810/91.0930 | (0.840 s), (0.860 s), (2.150 dt. 13.460/4.710 Hz) |
| Cortisone | C_21_H_28_O_5_ | 53-06-5 | 162.9500/120.9500/  104.9800/93.0100 | (2.080-2.160 m), (2.870 d. 12.160 Hz) |
| Deoxycytidine | C_9_H_13_N_3_O_4_ | 951-77-9 | 168.0767/112.0505/95.0239 /69.0447 | (3.730-3.850 m) |
| Deoxyuridine | C_9_H_12_N_2_O_5_ | 951-78-0 | 155.0451/113.0345/85.0396 | (4.030-4.070 m) |
| D-Fructose 6-phosphate | C_6_H_13_O_9_P | 643-13-0 | 147.0657/61.0290/43.0184 | (4.230 t. 7.710 Hz) |
| D-Gluconic acid | C_6_H_12_O_7_ | 526-95-4 | 115.0290/68.7940/68.0420 | (3.736-3.786 m) |
| D-Glucono-1,5-lactone | C_6_H_10_O_6_ | 90-80-2 | 73.0290/59.0133/43.0184 | (3.740-3.780 m) |
| D-Glucosamine 6-phosphate | C_6_H_14_NO_8_P | 55722-81-1 | 98.9842/45.0335/43.0178 | (3.570-3.640 m) |
| D-Glucose 6-phosphate | C_6_H_13_O_9_P | 56-73-5 | 128.3000/127.2000/82.2000 | (3.270 dd. 9.210/7.990 Hz), (3.710 t. 9.540 Hz). (4.640 d. 7.990 Hz), (5.220 d. 3.750 Hz) |
| D-Glucuronate | C_6_H_10_O_7_ | 70021-34-0 | 159.0287/131.0338/  117.0182/105.0182 | (3.289 t. 8.620 Hz), (3.581 dd. 9.780/3.760 Hz) |
| D-Glutamine | C_5_H_10_N_2_O_3_ | 5959-95-5 | 101.0709/84.0443 | (3.760 t. 6.160 Hz) |
| D-Mannose | C_6_H_12_O_6_ | 3458-28-4 | 145.0495/103.0389/91.0389 | (3.564 t. 9.705 Hz) |
| D-Mannose 6-phosphate | C_6_H_13_O_9_P | 3672-15-9 | 145.0501/122.9847/  103.0395/101.0239 | (3.434/3.447 d. 9.190 Hz) |
| D-Sorbitol | C_6_H_14_O_6_ | 50-70-4 | 61.1690/57.1960/43.3070 | (3.721 d. 2.88 Hz), (3.734-3.795 m), (3.813 d. 2.930 Hz) |
| dTMP | C_10_H_15_N_2_O_8_P | 365-07-1 | 127.0000/81.0000 | (1.910 s) |
| D-Xylose | C_5_H_10_O_5_ | 58-86-6 | 115.0389/91.0389 | (3.210 dd. 9.330/7.900 Hz), (3.420 t. 9.250 Hz), (3.510 dd. 9.360/3.690 Hz) |
| Estradiol-17beta | C_18_H_24_O_2_ | 50-28-2 | 107.0000/79.0820 | (2.030 br,s.), (2.210 br,s.), (3.510 s) |
| Estrone | C_18_H_24_O_3_ | 50-27-1 | 107.1990/77.3290 | (1.170-1.330 m), (3.290 t. 5.230 Hz) |
| Estriol | C_18_H_22_O_2_ | 53-16-7 | 105.0000/91.0000/55.0000 | (1.289-1.347 m) |
| Ethanolamine | C_2_H_7_NO | 141-43-5 | 62.0000/44.0494 | (3.130 d. 5.210 Hz), (3.810 d. 5.290 Hz) |
| Etiocholanolone | C_19_H_30_O_2_ | 53-42-9 | 133.6680/118.9600/  104.9940/90.4100 | (0.980 s), (2.030-2.120 m) |
| Riboflavin monophosphate | C_17_H_21_N_4_O_9_P | 146-17-8 | 255.0876/237.0770/  212.0818 | (2.390 s). (2.520 s) |
| Folate | C_19_H_19_N_7_O_6_ | 59-30-3 | 350.1359/295.0938/  267.0988 | (1.990-2.210 m) |
| gamma-L-Glutamyl-L-cysteine | C_8_H_14_N_2_O_5_S | 636-58-8 | 187.0535/159.0586/  122.0270 | (2.920-3.010 m) |
| Glutathione | C_10_H_17_N_3_O_6_S | 70-18-8 | 228.0978/187.0535/  159.0586 /102.0549 | (2.970 dd. 14.230/9.470 Hz), (3.740-3.820 m) |
| Glutathione disulfide | C_20_H_32_N_6_O_12_S_2_ | 27025-41-8 | 381.0903/177.0334/56.0500 | (2.1328-2.1694 m) |
| Glycerol | C_3_H_8_O_3_ | 56-81-5 | 75.0440/61.0284/57.0334/  31.0178 | (3.525-3.577 m) |
| Glycocholate | C_26_H_43_NO_6_ | 475-31-0 | 209.1328/76.0386 | (0.930 t. 7.390 Hz), (2.310-2.410 m), (3.880 m) |
| Homocarnosine | C_10_H_16_N_4_O_3_ | 3650-73-5 | 178.0974/139.0502/  108.0556 | (2.890-2.970 m) |
| Hydroxyproline | C_5_H_9_NO_3_ | 51-35-4 | 114.0549/96.0443 | (2.390-2.450 m), (3.460 dd. 12.640/3.870 Hz) |
| Lactose | C_12_H_22_O_11_ | 63-42-3 | 85.0670/61.1870 | (3.280 t. 8.600 Hz), (3.590 d. 3.680 Hz) |
| L-Arabinose | C_5_H_10_O_5_ | 7296-56-2 | 73.0284/61.0284/59.0128 | (3.820 dd. 9.780/3.570 Hz), (3.950 m) |
| L-Arabitol | C_5_H_12_O_5_ | 7643-75-6 | 135.0651/117.0546/75.0440 | (3.630-3.690 m), (3.910-3.950 m) |
| Lathosterol | C_27_H_46_O | 80-99-9 | 327.3052/71.0861/57.0704 | (0.920 d. 6.550 Hz), (1.000 t. 9.460 Hz), (1.200 q. 9.240 Hz) |
| L-Cystathionine | C_7_H_14_N_2_O_4_S | 56-88-2 | 177.0692/160.0426/  148.0426 | (3.950 dt. 7.110/4.410 Hz) |
| L-Glutamate | C_5_H_9_NO_4_ | 56-86-0 | 102.0549/84.0443 | (3.748 dd. 7.186/4.724 Hz) |
| L-Gulono-1,4-lactone | C_6_H_10_O_6_ | 1128-23-0 | 57.0341/45.0343/41.0393 | (3.784-3.823 m) |
| L-Hydroxylysine | C_6_H_14_N_2_O_3_ | 1190-94-9 | 82.0000/74.0000/67.0000 | (3.730-3.810 m), (3.840-3.940 m) |
| L-Isoleucine | C_6_H_13_NO_2_ | 73-32-5 | 114.0913/86.0964 | (0.997 d. 7.001 Hz) |
| L-Methionine | C_5_H_11_NO_2_S | 63-68-3 | 104.0528/88.0215 | (2.055-2.241 m) |
| L-Palmitoylcarnitine | C_23_H_46_NO_4_ | 2364-67-2 | 85.0293/60.0820 | (2.690 d. 6.410 Hz), (3.700 d. 13.820 Hz) |
| L-Proline | C_5_H_9_NO_2_ | 147-85-3 | 98.0600/72.0807 | (4.120 dd. 8.630/6.420 Hz) |
| Maltose | C_12_H_22_O_11_ | 69-79-4 | 244.3300/143.5900/93.8600/68.7200 | (3.410 t. 9.550 Hz), (3.930 d. 2.380 Hz), (3.620 m) |
| myo-Inositol | C_6_H_12_O_6_ | 87-89-8 | 163.0600/145.0495/  101.0233 | (3.268 t. 9.344 Hz), (3.613 t. 9.702 Hz), (4.053 t. 2.839 Hz) |
| N6-(L-1,3-Dicarboxypropyl)-L-lysine | C_11_H_20_N_2_O_6_ | 997-68-2 | 185.1290/84.0813 | (2.070 q. 7.140 Hz), (2.390 t. 6.840 Hz), (3.750 t. 6.090 Hz) |
| N-Acetyl-D-mannosamine | C_8_H_15_NO_6_ | 7772-94-3 | 96.0000/84.0000/69.0000 | (2.040 s), (2.080 s), (3.520 t. 9.880 Hz) |
| N-Acetyl-L-glutamate | C_7_H_11_NO_5_ | 1188-37-0 | 144.0655/130.0498/  126.0549 /102.0549/100.0393 | (2.008-2.077 m) |
| Pregnenolone | C_21_H_32_O_2_ | 145-13-1 | 259.2062/239.1800/  225.1643/41.0391 | (1.447-1.507 m) |
| Progesterone | C_21_H_30_O_2_ | 57-83-0 | 109.2190/97.2650/79.3320 | (1.200 s), (1.430-1.500 m), (2.030 dd. 4.800/3.290 Hz), (2.540 t. 8.990 Hz) |
| Prostaglandin D2 | C_20_H_32_O_5_ | 41598-07-6 | 137.0603/71.0861/67.0548 | (2.440 dd) |
| Prostaglandin E2 | C_20_H_32_O_5_ | 363-24-6 | 137.0603/67.0548/41.0391 | (2.170 t), (3.910-3.980 m) |
| Raffinose | C_18_H_32_O_16_ | 512-69-6 | 325.1129/181.0706/  163.0600 /93.0546 | (4.120 d. 6.300 Hz) |
| S-Adenosyl-L-homocysteine | C_14_H_20_N_6_O_5_S | 979-92-0 | 220.0638/136.0617/  119.0352 | (2.630 t. 7.730 Hz), (2.800 dd. 13.730/6.900 Hz), (2.920 dd. 13.730/6.080 Hz), (4.140 t) |
| sn-Glycero-3-phosphocholine | C_8_H_20_NO_6_P | 28319-77-9 | 98.9839/80.9738 | (3.200 s) |
| sn-Glycerol 3-phosphate | C_3_H_9_O_6_P | 57-03-4 | 99.0000/81.0000 | (3.670 dd. 11.800/4.630 Hz) |
| Sphinganine | C_18_H_39_NO_2_ | 764-22-7 | 60.2170/55.5380/55.2850 | (1.220-1.370 m) |
| Sphingomyelin | C_41_H_83_N_2_O_6_P | 58909-84-5 | 664.5252/540.5326 | (1.190-1.340 m), (3.540 m) |
| Sphingosine | C_18_H_37_NO_2_ | 123-78-4 | 95.0850/93.0692/83.0484 | (2.070 q), (3.480 dd), (3.670 dd. 10.840/4.460 Hz), (3.960 t) |
| Stachyose | C_24_H_42_O_21_ | 470-55-3 | 649.2185/487.1657/  325.1129 /181.0706/163.0600 | (3.960-4.010 m) |
| Sucrose | C_12_H_22_O_11_ | 57-50-1 | 181.0706/163.0600/  121.0495/105.0546 | (3.460 t. 9.300 Hz), (3.670 s), (4.210 d. 8.750 Hz) |
| Taurocholate | C_26_H_45_NO_7_S | 81-24-3 | 319.2415/199.1484/  126.0224 | (2.029-2.088 m) |
| Testosterone | C_19_H_28_O_2_ | 58-22-0 | 109.0420/97.0810/79.1180 | (3.614-3.708 m) |
| Tetrahydrofolate | C_19_H_23_N_7_O_6_ | 135-16-0 | 339.7790/188.8500/  166.9260/73.9860 | (3.170-3.230 m), (3.510 d. 11.060 Hz) |
| Thymidine | C_10_H_14_N_2_O_5_ | 50-89-5 | 169.0607/127.0502/  109.0396/97.0396/82.0287 | (2.366 dd. 6.630/5.530 Hz) |
| UDP-glucose | C_15_H_24_N_2_O_17_P_2_ | 133-89-1 | 386.9989/227.0662/  190.9505 /113.0345/83.0239 | (3.740-3.800 m) |
| UDP-N-acetyl-alpha-D-glucosamine | C_17_H_27_N_3_O_17_P_2_ | 528-04-1 | 539.0673/364.0193/  284.0529 /113.0345/83.0239 | (2.070 s) |
| Vitamin D3 | C_27_H_44_O | 67-97-0 | 285.2582/57.0704/55.0548 | (1.010 q. 9.020 Hz), (2.350-2.460 m), (2.570 dd. 12.960/2.720 Hz) |

**Table S2**: Metabolites of control HaCaT and treated with 0.1 mM and 0.3 mM DCBQ-OH; + denotes active metabolites in the sample, while - denotes non present metabolites.

| **Metabolites** | **Control** | **0.1 mM DCBQ-OH** | **0.3 mM DCBQ-OH** |
| --- | --- | --- | --- |
| (9Z,12Z,15Z)-Octadecatrienoic acid | - | - | + |
| 11-Deoxycorticosterone | + | + | + |
| 17alpha,20alpha-Dihydroxypregn-4-en-3-one | + | + | + |
| 17alpha-Hydroxypregnenolone | + | + | + |
| 17alpha-Hydroxyprogesterone | + | + | - |
| 2-Hydroxyestradiol | + | + | + |
| 2-Hydroxyestrone | + | + | + |
| 2-Methoxy-17beta-estradiol | + | + | + |
| 2-Methoxyestrone | + | + | + |
| 5alpha-Androstane-3,17-dione | + | + | + |
| 5-Oxo-D-proline | + | + | - |
| 7-Dehydrocholesterol | + | + | + |
| Aldosterone | + | + | + |
| alpha-D-Galactose | + | + | - |
| alpha-D-Glucose | + | + | - |
| Androstenedione | + | + | + |
| beta-D-Fructose | + | + | + |
| beta-Sitosterol | + | + | + |
| Biotin | - | + | - |
| Chenodeoxycholate | + | + | + |
| Cholesterol sulfate | + | + | + |
| Corticosterone | + | + | + |
| Cortisone | + | + | + |
| D-Glucosamine 6-phosphate | + | - | - |
| D-Glucuronate | - | + | - |
| D-Glucose | + | + | - |
| D/L-Glutamine | + | + | + |
| D-Mannose | + | + | + |
| D-Sorbitol | + | + | - |
| dTMP | - | + | - |
| D-Xylose | + | + | + |
| Estradiol-17beta | + | + | + |
| Estrone | + | - | + |
| Estriol | + | - | - |
| Etiocholanolone | + | + | + |
| Folate | + | + | + |
| gamma-L-Glutamyl-L-cysteine | - | + | - |
| Glutathione | - | + | - |
| Glutathione disulfide | - | + | - |
| Glycerol | + | + | + |
| Glycocholate | + | + | + |
| Homocarnosine | - | + | - |
| Hydroxyproline | - | + | + |
| Lactose | + | + | + |
| L-Arabitol | + | - | - |
| Lathosterol | + | + | + |
| L-Glutamate | + | + | + |
| L-Methionine | - | - | + |
| L-Hydroxylysine | + | + | - |
| L-Proline | + | + | + |
| Maltose | + | + | - |
| N-Acetyl-L-glutamate | - | - | + |
| N6-(L-1,3-Dicarboxypropyl)-L-lysine | + | + | - |
| Pregnenolone | + | + | + |
| Progesterone | + | + | + |
| Prostaglandin D2 | + | + | + |
| Prostaglandin E2 | + | + | + |
| Raffinose | + | + | - |
| S-Adenosyl-L-homocysteine | - | + | + |
| Sphinganine | - | - | + |
| Sphingomyelin | + | + | + |
| Sphingosine | + | - | + |
| Stachyose | + | + | - |
| Taurocholate | + | + | + |
| Testosterone | + | + | + |
| Tetrahydrofolate | + | + | + |
| Vitamin D3 | + | + | + |

**Table S3**: Metabolites of control HaCaT and treated with 0.05 mM and 0.075 mM DCBQ; + denotes active metabolites in the sample, while - denotes non present metabolites.

| **Metabolites** | **control** | **0.05 mM DCBQ** | **0.075 mM DCBQ** |
| --- | --- | --- | --- |
| (R)-Lipoate | - | + | + |
| 11-Deoxycorticosterone | + | + | + |
| 17alpha,20alpha-Dihydroxypregn-4-en-3-one | + | + | + |
| 17alpha-Hydroxypregnenolone | + | + | + |
| 17alpha-Hydroxyprogesterone | - | - | + |
| 2-Hydroxyestradiol | - | + | + |
| 2-Hydroxyestrone | - | + | + |
| 2-Methoxy-17beta-estradiol | - | + | + |
| 2-Methoxyestrone | + | + | + |
| 5alpha-Androstane-3,17-dione | + | + | + |
| 7-Dehydrocholesterol | + | + | + |
| Aldosterone | + | + | + |
| alpha,alpha-Trehalose | + | + | + |
| alpha-D-Galactose | + | + | + |
| alpha-D-Galactose 1-phosphate | + | + | + |
| alpha-D-Glucose | + | + | + |
| alpha-D-Glucose 1,6-bisphosphate | - | - | + |
| Androstenedione | + | + | + |
| beta-Alanyl-N(pi)-methyl-L-histidine | - | + | - |
| beta-D-Fructose | + | + | + |
| beta-D-Fructose 2,6-bisphosphate | - | - | + |
| beta-D-Glucose 6-phosphate | + | + | + |
| beta-Sitosterol | + | + | + |
| Biotin | + | - | - |
| Chenodeoxycholate | + | + | + |
| Cholesterol ester | + | + | + |
| Cholesterol sulfate | + | + | + |
| CoA | - | + | - |
| Corticosterone | - | + | + |
| Cortisone | - | + | + |
| Deoxycytidine | - | - | + |
| Deoxyuridine | - | - | + |
| D-Fructose 6-phosphate | + | + | + |
| D-Gluconic acid | - | - | + |
| D-Glucono-1,5-lactone | - | - | + |
| D-Glucosamine 6-phosphate | + | - | + |
| D-Glucose | + | + | + |
| D-Glucose 6-phosphate | + | + | + |
| D-Glucuronate | + | + | + |
| D-Glutamine | - | + | + |
| D-Mannose | + | + | + |
| D-Mannose 6-phosphate | + | + | + |
| D-Sorbitol | + | + | + |
| dTMP | + | + | + |
| D-Xylose | + | + | + |
| Estradiol-17beta | - | + | - |
| Ethanolamine | - | + | + |
| Etiocholanolone | + | + | + |
| FMN | - | + | + |
| Glutathione | - | + | - |
| Glycerol | - | - | + |
| Glycocholate | + | + | + |
| Hydroxyproline | - | + | + |
| Lactose | + | + | + |
| L-Arabinose | + | + | + |
| L-Arabitol | - | + | + |
| Lathosterol | + | + | - |
| L-Cystathionine | - | + | + |
| L-Glutamate | - | - | + |
| L-Glutamine | - | + | + |
| L-Gulono-1,4-lactone | + | + | + |
| L-Hydroxylysine | + | + | - |
| L-Palmitoylcarnitine | - | - | + |
| L-Isoleucine | + | - | - |
| L-Proline | + | - | + |
| Maltose | + | + | + |
| N6-(L-1,3-Dicarboxypropyl)-L-lysine | + | - | - |
| myo-Inositol | - | - | + |
| N-Acetyl-D-mannosamine | + | + | + |
| Pregnenolone | + | + | + |
| Progesterone | + | + | + |
| Prostaglandin D2 | - | + | + |
| Prostaglandin E2 | + | + | + |
| Raffinose | + | + | + |
| S-Adenosyl-L-homocysteine | - | + | - |
| sn-Glycero-3-phosphocholine | - | + | + |
| sn-Glycerol 3-phosphate | + | + | + |
| Sphinganine | + | + | + |
| Sphingomyelin | + | + | - |
| Sphingosine | + | + | + |
| Stachyose | + | + | + |
| Sucrose | + | + | + |
| Taurocholate | + | + | + |
| Testosterone | + | + | + |
| Tetrahydrofolate | - | + | + |
| Thymidine | - | - | + |
| UDP-glucose | - | + | + |
| UDP-N-acetyl-alpha-D-glucosamine | - | + | - |
| Vitamin D3 | + | + | + |

**Table S4**: Relative quantification values of the metabolites of HaCaT cells (control and treated with 0.1 mM and 0.3 mM of DCBQ-OH) ± standard deviation (n=3); NQ: non-quantifiable.

| **Metabolites** | **Control** | **0.1 mM DCBQ-OH (IC_20_)** | **0.3 mM DCBQ-OH (IC_50_)** |
| --- | --- | --- | --- |
| 11-Deoxycorticosterone | 0.082 ± 0.005 | 0.146 ± 0.006 | NQ |
| 17alpha,20alpha-Dihydroxypregn-4-en-3-one | 0.082 ± 0.004 | 0.144 ± 0.006 | 0.062 ± 0.003 |
| 17alpha-Hydroxypregnenolone | 0.109 ± 0.005 | 0.161 ± 0.007 | 0.063 ± 0.004 |
| 2-Hydroxyestradiol | 0.057 ± 0.003 | 0.129 ± 0.006 | 0.048 ± 0.002 |
| 2-Hydroxyestrone | 0.051 ± 0.003 | 0.078 ± 0.004 | 0.024 ± 0.001 |
| 2-Methoxy-17beta-estradiol | NQ | NQ | NQ |
| 2-Methoxyestrone | NQ | NQ | NQ |
| 5alpha-Androstane-3,17-dione | 0.065 ± 0.003 | 0.107 ± 0.006 | 0.045 ± 0.002 |
| 7-Dehydrocholesterol | 0.015 ± 0.007 | 0.037 ± 0.002 | 0.011 ± 0.001 |
| Aldosterone | 0.088 ± 0.05 | 0.152 ± 0.008 | 0.087 ± 0.005 |
| Androstenedione | 0.012 ± 0.001 | 0.045 ± 0.002 | 0.006 ± 0.001 |
| beta-D-Fructose | NQ | NQ | NQ |
| beta-Sitosterol | 0.014 ± 0.001 | 0.027 ± 0.001 | 0.005 ± 0.001 |
| Chenodeoxycholate | 0.020 ± 0.001 | 0.041 ± 0.002 | NQ |
| Cholesterol sulfate | 0.363 ± 0.015 | 0.208 ± 0.01 | 0.108 ± 0.006 |
| Corticosterone | 0.074 ± 0.003 | 0.116 ± 0.01 | 0.044 ± 0.002 |
| Cortisone | 0.016 ± 0.001 | 0.029 ± 0.004 | 0.009 ± 0.001 |
| D/L-Glutamine | 0.065 ± 0.003 | 0.169 ± 0.002 | 0.086 ± 0.005 |
| D-Mannose | 0.045 ± 0.002 | 0.082 ± 0.003 | 0.017 ± 0.001 |
| D-Xylose | 0.015 ± 0.001 | 0.048 ± 0.005 | 0.014 ± 0.001 |
| Estradiol-17beta | 0.035 ± 0.002 | 0.051 ± 0.02 | 0.016 ± 0.001 |
| Etiocholanolone | 0.085 ± 0.005 | 0.113 ± 0.001 | 0.121 ± 0.006 |
| Folate | 0.47 ± 0.02 | 0.279 ± 0.002 | 0.084 ± 0.005 |
| Glycerol | 0.025 ± 0.001 | 0.053 ± 0.006 | 0.018 ± 0.001 |
| Glycocholate | 0.029 ± 0.001 | 0.028 ± 0.002 | NQ |
| Lactose | 0.040 ± 0.002 | 0.054 ± 0.004 | NQ |
| Lathosterol | 0.185 ± 0.008 | 0.099 ± 0.006 | NQ |
| L-Glutamate | 0.015 ± 0.001 | 0.052 ± 0.002 | NQ |
| L-Proline | 0.043 ± 0.002 | 0.087 ± 0.004 | 0.028 ± 0.002 |
| Pregnenolone | NQ | NQ | NQ |
| Progesterone | 0.128 ± 0.006 | 0.188 ± 0.011 | 0.076 ± 0.003 |
| Prostaglandin D2 | NQ | NQ | NQ |
| Prostaglandin E2 | 0.089 ± 0.004 | 0.195 ± 0.008 | 0.066 ± 0.003 |
| Sphingomyelin | 0.33 ± 0.02 | 0.520 ± 0.03 | 0.276 ± 0.01 |
| Taurocholate | NQ | NQ | NQ |
| Testosterone | 0.072 ± 0.003 | 0.125 ± 0.006 | 0.077 ± 0.004 |
| Tetrahydrofolate | 0.049 ± 0.002 | 0.101 ± 0.004 | 0.042 ± 0.002 |
| Vitamin D3 | 0.022 ± 0.001 | 0.041 ± 0.002 | 0.019 ± 0.001 |

**Table S5**: Relative quantification values of the metabolites of HaCaT cells (control and treated with 0.05 mM and 0.075 mM of DCBQ) ± standard deviation (n=3); NQ: non-quantifiable.

| **Metabolites** | **control** | **0.05 mM DCBQ (IC_20_)** | **0.075 mM DCBQ (IC_50_)** |
| --- | --- | --- | --- |
| 11-Deoxycorticosterone | 0.135 ± 0.006 | 0.177 ± 0.007 | 0.039 ± 0.002 |
| 17alpha,20alpha-Dihydroxypregn-4-en-3-one | 0.072 ± 0.003 | 0.085 ± 0.005 | 0.061 ± 0.003 |
| 17alpha-Hydroxypregnenolone | 0.043 ± 0.002 | 0.057 ± 0.002 | 0.057 ± 0.003 |
| 2-Methoxyestrone | NQ | NQ | NQ |
| 5alpha-Androstane-3,17-dione | 0.23 ± 0.01 | 0.069 ± 0.004 | 0.085 ± 0.004 |
| 7-Dehydrocholesterol | NQ | 0.102 ± 0.006 | 0.115 ± 0.007 |
| Aldosterone | 0.078 ± 0.004 | 0.084 ± 0.003 | 0.062 ± 0.002 |
| alpha.alpha-Trehalose | 0.106 ± 0.004 | 0.111 ± 0.006 | NQ |
| alpha-D-Galactose | NQ | 0.09 ± 0.004 | 0.104 ± 0.005 |
| alpha-D-Galactose 1-phosphate | NQ | NQ | NQ |
| alpha-D-Glucose | 0.25 ± 0.01 | 0.978 ± 0.06 | 0.519 ± 0.02 |
| Androstenedione | 0.121 ± 0.005 | 0.204 ± 0.01 | NQ |
| beta-D-Fructose | 0.0403 ± 0.002 | 0.091 ± 0.005 | 0.061 ± 0.003 |
| beta-D-Glucose 6-phosphate | NQ | NQ | NQ |
| beta-Sitosterol | 0.071 ± 0.004 | 0.099 ± 0.004 | 0.056 ± 0.003 |
| Chenodeoxycholate | 0.056 ± 0.002 | 0.033 ± 0.002 | 0.026 ± 0.001 |
| Cholesterol ester-CE(12:0) | NQ | NQ | NQ |
| Cholesterol sulfate | NQ | 0.049 ± 0.002 | 0.035 ± 0.002 |
| D-Fructose 6-phosphate | NQ | 0.187 ± 0.01 | NQ |
| D-Glucose | 0.25 ± 0.01 | 0.978 ± 0.05 | 0.519 ± 0.03 |
| D-Glucose 6-phosphate | 0.24 ± 0.01 | 0.19 ± 0.01 | 0.161 ± 0.008 |
| D-Glucuronate | 0.048 ± 0.002 | 0.049 ± 0.002 | NQ |
| D-Mannose | NQ | 0.101 ± 0.006 | 0.068 ± 0.003 |
| D-Mannose 6-phosphate | 0.063 ± 0.003 | 0.067 ±0.004 | 0.038 ± 0.002 |
| D-Sorbitol | 0.094 ± 0.006 | 0.101 ± 0.005 | 0.088 ± 0.005 |
| dTMP | NQ | NQ | 0.591 ± 0.02 |
| D-Xylose | 0.088 ± 0.004 | 0.106 ± 0.006 | 0.031 ± 0.001 |
| Etiocholanolone | NQ | NQ | NQ |
| Glycocholate | 0.106 ± 0.006 | 0.152 ± 0.008 | NQ |
| Lactose | 0.025 ± 0.001 | 0.070 ± 0.004 | 0.035 ± 0.001 |
| L-Arabinose | 0.068 ± 0.003 | 0.053 ± 0.003 | 0.035 ± 0.002 |
| L-Gulono-1,4-lactone | NQ | NQ | NQ |
| Maltose | 0.124 ± 0.006 | 0.117 ± 0.007 | 0.057 ± 0.002 |
| N-Acetyl-D-mannosamine | 0.070 ± 0.003 | 0.077 ± 0.003 | NQ |
| Pregnenolone | NQ | NQ | NQ |
| Progesterone | 0.069 ± 0.003 | 0.045 ± 0.002 | 0.048 ± 0.002 |
| Prostaglandin E2 | 0.053 ± 0.003 | 0.049 ± 0.002 | 0.019 ± 0.001 |
| Raffinose | NQ | NQ | 0.130 ± 0.007 |
| sn-Glycerol 3-phosphate | 0.117 ± 0.006 | 0.189 ± 0.009 | NQ |
| Sphinganine | 0.126 ± 0.006 | 0.130 ± 0.005 | NQ |
| Sphingosine | 0.076 ± 0.003 | 0.081 ± 0.004 | NQ |
| Stachyose | 0.092 ± 0.005 | 0.067 ± 0.003 | NQ |
| Sucrose | 0.215 ± 0.01 | 0.148 ± 0.007 | 0.062 ± 0.003 |
| Taurocholate | NQ | NQ | NQ |
| Testosterone | 0.168 ± 0.01 | 0.20 ± 0.01 | NQ |
| Vitamin D3 | 0.069 ± 0.004 | 0.095 ± 0.004 | 0.027 ± 0.001 |

**References**

Chatzimitakos, T. G., Kasouni, A. I., Troganis, A. N., & Stalikas, C. D. (2018). Carbonization of Human Fingernails: Toward the Sustainable Production of Multifunctional Nitrogen and Sulfur Codoped Carbon Nanodots with Highly Luminescent Probing and Cell Proliferative/Migration Properties [Research-article]. *ACS Applied Materials and Interfaces*, *10*(18), 16024–16032. https://doi.org/10.1021/acsami.8b03263

Hung, S., Mohan, A., Reckhow, D. A., & Godri Pollitt, K. J. (2019). Assessment of the in vitro toxicity of the disinfection byproduct 2,6-dichloro-1,4-benzoquinone and its transformed derivatives. *Chemosphere*, *234*, 902–908. https://doi.org/10.1016/j.chemosphere.2019.06.086

Wiley, J., & Sons. (2021). *Inc. SpectraBase; SpectraBase Compound ID=79hG9Xj6tLU SpectraBase Spectrum ID=sDuzxU6xI*. https://spectrabase.com/spectrum/sDuzxU6xI
